# Genetically Modified Hepatocytes Targeting Bilirubin and Ammonia Metabolism for the Construction of Bioartificial Liver System

**DOI:** 10.34133/bmr.0043

**Published:** 2024-07-15

**Authors:** Ke Wang, Yuankui Zhu, Mengqing Li, Yaxi Yang, Dianbao Zuo, Junfeng Sheng, Xinhai Zhang, Wei Wang, Ping Zhou, Mingqian Feng

**Affiliations:** ^1^ College of Biomedicine and Health, College of Life Science and Technology, Huazhong Agricultural University, Wuhan, Hubei 430070, China.; ^2^ School of Basic Medicine, Tongji Medical College, Huazhong University of Science and Technology, Wuhan, Hubei 430030, China.; ^3^ Wuhan TOGO Medical Technology Co. Ltd., Wuhan, Hubei 430205, China.

## Abstract

Acute liver failure (ALF) is a complex syndrome that impairs the liver’s function to detoxify bilirubin, ammonia, and other toxic metabolites. Bioartificial liver (BAL) aims to help ALF patients to pass through the urgent period by temporarily undertaking the liver’s detoxification functions and promoting the recovery of the injured liver. We genetically modified the hepatocellular cell line HepG2 by stably overexpressing genes encoding UGT1A1, OATP1B1, OTC, ARG1, and CPS1. The resulting SynHeps-II cell line, encapsulated by Cytopore microcarriers, dramatically reduced the serum levels of bilirubin and ammonia, as demonstrated both in vitro using patient plasma and in vivo using ALF animal models. More importantly, we have also completed the 3-dimensional (3D) culturing of cells to meet the demands for industrialized rapid and mass production, and subsequently assembled the plasma-cell contacting BAL (PCC-BAL) system to fulfill the requirements of preclinical experiments. Extracorporeal blood purification of ALF rabbits with SynHeps-II-embedded PCC-BAL saved more than 80% of the animals from rapid death. Mechanistically, SynHeps-II therapy ameliorated liver and brain inflammation caused by high levels of bilirubin and ammonia and promoted liver regeneration by modulating the nuclear factor κB (NF-κB) and signal transducer and activator of transcription 3 (STAT3) pathways. Also, SynHeps-II treatment reduced cerebral infiltration of neutrophils, reduced reactive oxygen species (ROS) levels, and mitigated hepatic encephalopathy. Taken together, SynHeps-II cell-based BAL was promising for the treatment of ALF patients and warrants clinical trials.

## Introduction

Liver is not only a toxin-metabolizing organ but also an immune organ enriched with innate immune cells such as resident macrophages (also known as Kupffer cells), innate lymphoid cells, and mucosa-associated invariant T (MAIT) cells [1]. Acute liver failure (ALF) is a syndrome of high risk and high mortality, characterized by rapidly progressive liver dysfunction and hepatocellular necrosis. ALF patients frequently develop systemic inflammatory response syndrome (SIRS) and vascular endothelial dysfunction, which collectively progress to multi-organ failure (MOF) [2–6]. ALF can be exacerbated by inducers of innate immune response, such as dysbiosis of the gut microbiota and gut leakage, activity of hepatitis viruses, and the release of damage-associated molecular patterns (DAMPs) from dying hepatocytes [1,4].

The molecular mechanisms underlying the pathogenesis of ALF depend on the primary etiology of liver injury and are influenced by the accumulation of toxic metabolites such as bilirubin and ammonia, which promote systemic inflammatory response [7–9]. UDP (uridine diphosphate) glucuronosyltransferase family 1 member A1 (UGT1A1) is the only enzyme capable of metabolizing bilirubin in the liver via glucuronic acid modification, which converts bilirubin into a nontoxic conjugated form that is then excreted from the body [10]. UGT1A1 dysfunction increases liver burden and aggravates hepatocyte damage during long-term bilirubin metabolism disorder [8]. Unconjugated bilirubin could activate nuclear factor κB (NF-κB) pathway and induce DNA damage in hepatocytes, leading to the significant increase of inflammatory factors [8]. ALF also impairs ammonia detoxification, leading to hyperammonemia and excessive activation of *N*-methyl-d-aspartate (NMDA) receptors in the brain, which contributes to ALF-induced hepatic encephalopathy [11]. Ammonia not only is directly toxic to astrocytes but also induces neutrophil dysfunction with the release of reactive oxygen species (ROS), which contribute to oxidative stress and systemic inflammation of the brain [12]. Dialysis is one of the clinical treatment options for rapid ammonia removal and can be performed by hemodialysis or continuous renal replacement therapy (CRRT) [9]. Clinical trial with BAL therapy using C3A cell line, a subclone of hepatoma cell line HepG2, failed in treating severe alcoholic hepatitis [13]. C3A was defective in detoxifying ammonia due to the complete absence of expression of 2 enzymes in urea cycle, ornithine transcarbamylase (OTC) and arginase 1 (ARG1) [14]. Genetically modified C3A cells by overexpressing OTC and ARG1 restored urea cycle [15].

To date, the only definitive treatment for ALF is liver transplantation. Investigational new therapies include hepatocyte transplantation, tissue engineering, plasma exchange [6], and liver support systems [16], including adsorption-based mechanical artificial liver (MAL) devices and hepatocyte-based bioartificial liver (BAL) devices [17–19]. MALs function through adsorbents (activated carbon, ion exchange resins, porous biomaterials, etc.) to remove toxic molecules from the plasma, including bilirubin, bile acids, and some fatty acids. However, due to the poor selectivity of adsorption and lack of synthetic capacity to better promote liver regeneration, most MALs fail to improve the overall survival rate of ALF patients [20].

Significantly different from MALs, BAL carries functional hepatocytes that are assembled with specially designed bioreactors. A major challenge for BAL applications is the availability of hepatocytes with superior properties in terms of expansion efficiency and metabolic functions required by the liver. Initial attempts used primary human hepatocytes, which were limited by insufficient supply, high production costs, and low proliferation capacity [21]. Up to now, the following cell types have also been used in the BAL system, swine-derived hepatocytes [17], the hepatoblastoma cell line HepG2-derived C3A [19], immortalized human hepatocytes [16], and transdifferentiated human hepatocytes [22]. Porcine primary hepatocytes have been used in clinical trials of bioartificial liver support system (BLSS), Academic Medical Center (AMC)-BAL, but xenogeneic porcine hepatocytes are immunogenic and carry endogenous retroviruses with the risk of infecting humans [23]. The hepatoblastoma cell line C3A was used in the extracorporeal cellular therapy (ELAD) system, and although the C3A cells secrete the required albumin (ALB), they lack the function of bilirubin metabolism and ammonia detoxification [24]. The ELAD system failed phase III clinical trials [13]. Immortalized cells are generally produced by lentiviral transduction of oncogenic genes such as human papillomavirus (HPV) E6/E7 and simian virus 40 large T-antigen (SV40T) into normal hepatocyte [16,25]. Immortalized cells may potentially have the tumorigenic risk of the lentivirus and the loss of function and karyotype changes after multiple passages [26]. Human induced pluripotent stem cells (hiPSCs) have the ability of self-renewal, proliferation, and differentiation and can be induced to differentiate into hepatocytes [27,28]. However, the complex differentiation process and the large number of 10^10^ to 10^11^ cells required for clinical therapy make the production of hiPSC hepatocytes extremely costly and time consuming [29].

**Fig. 1.**
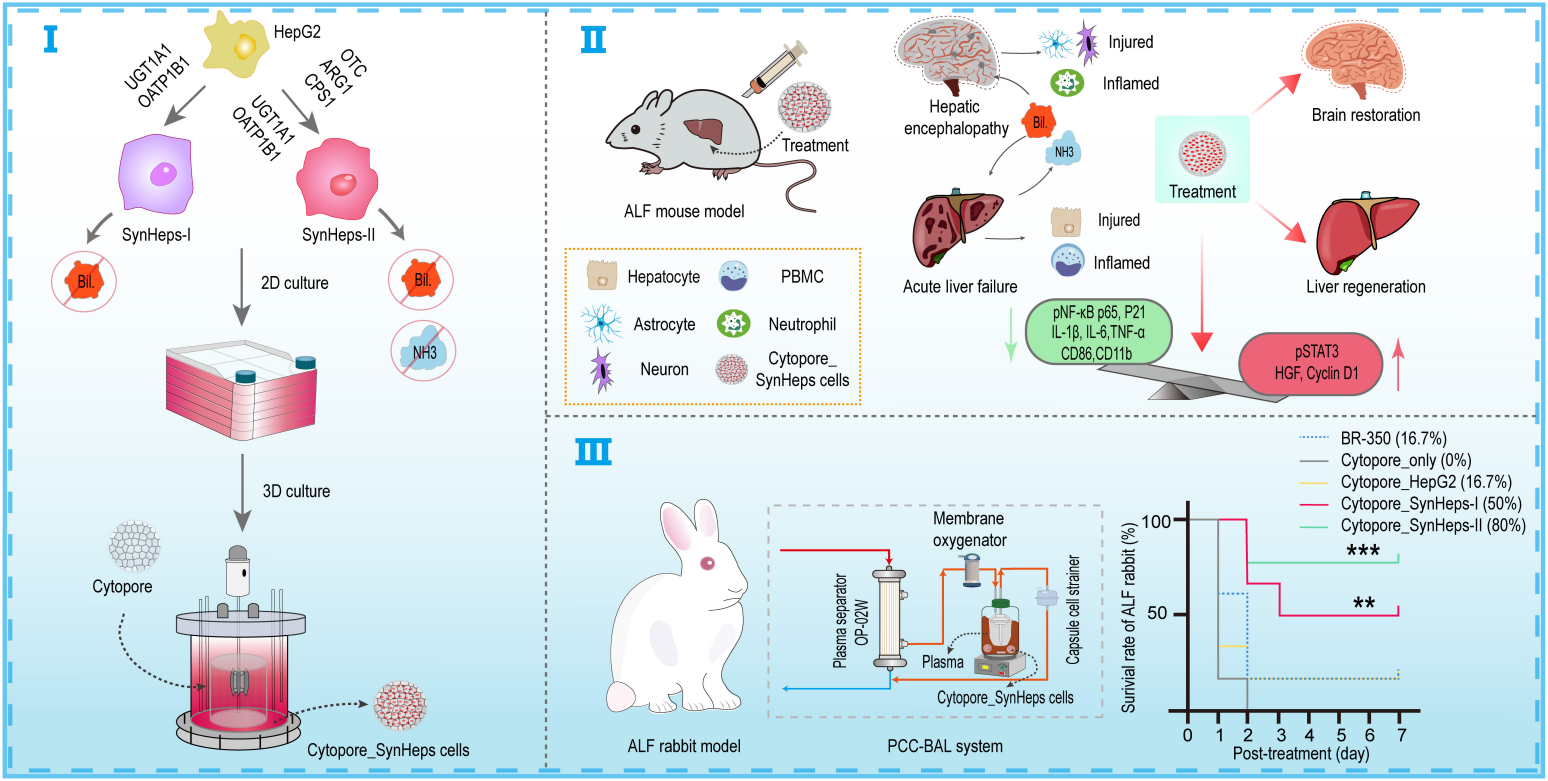
Outline of current study.

In the current study, we engineered HepG2 cells to make BAL. The whole study was outlined in Fig. 1. Considering its poor metabolic ability for bilirubin and ammonia, we stably transfected HepG2 cells with UGT1A1 and OATP1B1 (solute carrier organic anion transporter family member 1B1) genes, and the resulting SynHeps-I cell line gained the capacity to detoxify bilirubin by glucuronidation. SynHeps-I cells were further engineered by stably overexpressing OTC, ARG1, and CPS1 (carbamoyl-phosphate synthetase 1) genes to convert toxic ammonia to urea, resulting in a cell line named SynHeps-II. Surprisingly, compared to SynHeps-I and parental HepG2 cells, SynHeps-II cells not only metabolized bilirubin and ammonia efficiently but also were able to more effectively reduce proinflammatory cytokines, as demonstrated in the animal studies. Large-scale culturing of SynHeps-II cells in the 3-dimensional (3D) microcarrier Cytopore and embedding of Cytopore_cell complexes into specially designed bioreactors resulted in a new plasma-cell contacting BAL (PCC-BAL) system in which plasma was exposed to cells of the BAL. PCC-BAL showed impressive efficacy, rescuing approximately 80% of rabbits with ALF from rapid death.

## Materials and Methods

### Cell lines

Hepatic cell lines HepG2, Huh7, and human embryonic kidney cell line 293F were maintained as adherent monolayer cultures in Dulbecco’s modified Eagle’s medium (DMEM) (Thermo Fisher, Carlsbad, CA) supplemented with 10% fetal bovine serum (FBS) (AusGenex, Queensland, Australia), 1% l-glutamine, and 1% penicillin/streptomycin (Thermo Fisher, Carlsbad, CA) and incubated in 5% CO_2_ with a balance of air at 37 °C. Cells were passaged every 3 days and replaced with fresh medium. Both SynHeps-I and SynHeps-II cells were maintained in DMEM as parental HepG2 cells.

### Construction of SynHeps cell lines

To generate the SynHeps-I cell line, 3 × 10^6^ HepG2 cells were seeded in T-75 cell culture flask and transfected with 15 μg of linearized plasmid pTBSCH1-UGT1A1-OATP1B1 that was mixed with transfection reagent Lipofectamine 3000 (refer to the protocol for method and dosage) in 2 ml of Opti-MEM dilution medium (Thermo Fisher, Carlsbad, CA). Twenty-four hours after transfection, the cell culture medium was refreshed. Thereafter, the cells were passaged twice for expansion in order to get stably transformed cells. To isolate the stable clones, the expanded cells were digested and diluted with selection medium [DMEM containing 1.5 μg/ml puromycin (Thermo Fisher, Carlsbad, CA)] to 100 cells per ml, and 100 μl of cells was seeded in each well of a 96-well plate (average of 10 cells per well). The plate was incubated at 37 °C to allow formation of single colony, which was monitored with an inverted microscope (Olympus, IX83) every day. Wells with apparently one single colony were harvested for further expansion of the cells in a 24-well plate, and then in T-25 cell culture flask and T-75 cell culture flask. After 10 generations of passage in selection medium, the selection pressure was raised to 2.0 μg/ml of puromycin and continued to culture the colonies for additional 20 generations. The stable cell line with the highest bilirubin detoxification capacity was selected for the study and named SynHeps-I.

SynHeps-II cell line was generated in a similar way. Briefly, SynHeps-I cells were transfected with linearized plasmid pLBFCH2-OTC-ARG1-CPS1, and the selection medium was DMEM containing 4.5 μg/ml blasticidin (Thermo Fisher, Carlsbad, CA). After 10 generations of passage in selection medium, the selection pressure was raised to 5 μg/ml of blasticidin and continued to culture the colonies for additional 20 generations. Finally, after 30 generations of passage since single-colony formation, the stable cell line with the highest ammonia detoxification capacity was selected and named SynHeps-II.

### 3D culturing of the cell with Cytopore

The Cytopore microcarriers (GE, Boston, MA) were washed repeatedly with phosphate-buffered saline (PBS) and soaked overnight, followed by autoclaving at 121 °C for 30 min, after which the beads were allowed to settle naturally. These microparticles were then transferred to a 500-ml glass bottle containing 400 ml of DMEM and stored at 4 °C before use. To form Cytopore-encapsulated cell complex, the HepG2, SynHeps-I, and SynHeps-II cells were dispersed in the culture medium at a density of 1 × 10^6^ cells/ml to infiltrate the Cytopore microcarriers (2 × 10^9^ cells for 2.5 g of Cytopore). Cytopore_cell complexes were transferred to shaking flask containing complete DMEM. Cellular spheres formed 48 h after shaking horizontally at 60 rpm, 37 °C, and 5% CO_2_. Alternatively, the Cytopore_cell complexes were transferred to a fermenter containing complete DMEM, replenished halfway with glucose (GLC) and the necessary gas components to achieve a 5-day in-place culturing. The optical images of Cytopore-encapsulated cells as well as images of Cytopore_cell complexes stained with crystal violet (Sangon Biotech, Shanghai, China) were observed using an inverted microscope (Olympus, IX83). In addition, fluorescence images of Cytopore-encapsulated SynHeps-II cells carrying a green fluorescent protein (GFP) reporter gene were observed by fluorescence microscopy (Leica, SP8).

### Quantitative real-time PCR

Total RNA was extracted from cells and the liver tissue with TRIzol reagent (Thermo Fisher, Carlsbad, CA) according to the manufacturer′s instruction. Total cellular RNA was reverse-transcribed using Moloney murine leukemia virus (MMLV) reverse transcriptase (Thermo Fisher, Carlsbad, CA). The expression level of genes was measured by real-time polymerase chain reaction (PCR) using TB SYBR Green Premix Ex Taq (Takara Bio, Shiga, Japan). The quantitative real-time PCR primer sequences of glyceraldehyde-3-phosphate dehydrogenase (GAPDH) (the reference) and other genes were listed in Table S1. The fold changes of the expression level of the candidate genes relative to the reference gene were calculated using the normalized expression (ΔCt) method with default threshold values using CFX Manager Software (Bio-Rad, Hercules, CA).

### Protein extraction and Western blot analysis

Protein was extracted from liver tissue and cells with radioimmunoprecipitation assay (RIPA) buffer (Beyotime, Shanghai, China) containing cocktail protease and phosphatase inhibitors (Beyotime, Shanghai, China). Protein concentration was determined by a BCA protein assay kit (Beyotime, Shanghai, China). Total proteins were electrophoresis separated on 10% reducing SDS-PAGE (polyacrylamide gel electrophoresis) gel at 90 V for 30 min and 120 V for 1 h and then electrotransferred onto a polyvinylidene difluoride (PVDF) membrane (Merck KGaA, Darmstadt, Germany) using the Bio-Rad blotting transfer system. The membranes were subsequently probed with rabbit monoclonal antibodies against β-actin, UGT1A1, OATP1B1, OTC, ARG1, and CPS1 from Proteintech (Wuhan, China) and P21, Cyclin D1, HGF, NF-κB p65, extracellular signal-regulated kinase 1/2 (ERK1/2), signal transducer and activator of transcription 3 (STAT3), phospho-NF-κB p65, phospho-ERK1/2, and phospho-STAT3 from ABclonal (Wuhan, China) at 4 °C overnight. Thereafter, the membrane was incubated with horseradish peroxidase (HRP)-conjugated goat anti-rabbit antibodies. Proteins were visualized using an enhanced chemiluminescence kit (Bio-Rad, Hercules, CA).

### ELISA

The serum concentrations of proinflammatory cytokines IL-1β, IL-6, and tumor necrosis factor-α (TNF-α) in mouse and rabbit were measured using enzyme-linked immunosorbent assay (ELISA) according to the protocol of the kits (Coibo bio, Shanghai, China). Human cytokines from peripheral blood mononuclear cells (PBMCs)/neutrophils treated with bilirubin/NH_4_Cl (Sigma-Aldrich, St. Louis, MO) were measured using corresponding assay kit from Mlbio (Shanghai, China).

### Histology, immunohistochemical, and immunofluorescence studies

Liver and brain tissues of the experimental animals were fixed in 10% buffered formalin, embedded in paraffin, and cut into 5-μm thickness sections. Specimens were stained with hematoxylin and eosin (H&E). Immunohistochemical and immunofluorescence staining was performed on formalin-fixed and paraffin-embedded tissue sections using corresponding antibodies for Caspase3, P21, Ki67, and hepatocyte growth factor (HGF) from Proteintech (Wuhan, China), CD86, CD163, CD31, and CD11b from ABclonal (Wuhan, China), and 4′,6-diamidino-2-phenylindole (DAPI) from Sangon Biotech (Shanghai, China).

### Serum biochemical testing

Patient plasma total bilirubin (TBIL), indirect bilirubin (IBIL), ammonia (NH3), ALB, and urea were quantified by clinical laboratory testing using automatic biochemical analyzers (Siemens, ADVIA 1800). Serum levels of the biochemical markers alanine aminotransferase (ALT), aspartate aminotransferase (AST), total bile acid (TBA), TBIL, blood urea nitrogen (BUN), and ammonia in mice and rabbits were determined with the corresponding kits from Njjcbio (Nanjing, China). In addition, blood gas-related indicators (Na^+^, K^+^, Ca^2+^, GLC, and pH) in rabbits were measured using a blood gas analyzer (Werfen, Bedford, MA).

### Measurement of intracellular ROS level

Dihydroethidium (DHE) fluorescent probes were used to detect intracellular ROS level in brain tissues (DHE probes from Sigma-Aldrich) and cells (ROS Detection Kit from Box Bio). For DHE staining, cryosections were incubated with 10 μM DHE at 37 °C for 30 min and then observed by confocal microscopy to determine the percentage of the DHE-stained area. For ROS detection in cultured cells, the cells were seeded in a 24-well plate and incubated with different concentrations of NH_4_Cl for 24 h. Fluorescence was detected by a multimode microplate reader (Tecan, Mannedorf, Switzerland) at 535-nm excitation and 610-nm emission.

### Collection of cerebrospinal fluid in mice

Cerebrospinal fluid (CSF) was collected in the cisterna magna of mice by a glass capillary (internal diameter, 1.30 mm). Mice were anesthetized by inhalation of isoflurane (2% to 3%) and then fixed with their heads 45° down at the brain localizer (RWD, Shenzhen, China). Surgical scissors were used to make an opening in the neck of the mice, and the surrounding muscles were carefully stripped to expose the cisterna magna and dry the surrounding blood. The glass capillary was carefully inserted into the cisterna magna at an angle of 45°. The needle was slowly rotated, after which the CSF will flow back out along the capillary. Five to 10 μl of CSF were withdrawn each time, after which the CSF was centrifuged at 2,000 rpm for 10 min at 4 °C, and the supernatant of the CSF was taken and used for the next step of the assay.

### Preparation of mouse primary hepatocytes

Mice were anesthetized with inhalation of isoflurane (at a dose of 2% to 3%), and the dose of isoflurane was maintained at 0.5% to 1.5% after the mice became unconscious. Subsequently, mouse hair was cut, and the abdominal cavity was opened on an ultra-clean bench to expose the hepatic portal vein and inferior vena cava. A 20-gauge indwelling needle was pushed into the inferior vena cava, and the mice were perfused with preheated (at 42 °C) Hanks’ balanced salt solution (HBSS) (without calcium and magnesium) containing 0.25 M EDTA (Sigma-Aldrich, St. Louis, MO) at a flow rate of 10 ml/min for 5 min. The perfusate was then flowed out of the open hepatic portal vein and then replaced with 30 ml of 0.05% collagenase IV (Thermo Fisher, Carlsbad, CA) in DMEM (without glutamine and supplemented with 5% FBS and 1% penicillin/streptomycin), during which the hepatic portal vein was intermittently clamped, which allowed the perfusate to take full effect in the liver. Afterward, the perfused mouse liver tissues were minced, abraded with perfusate (DMEM with 0.05% collagenase IV and 1% penicillin/streptomycin) at 4 °C for 10 min to make a hepatocyte suspension, passed through a 70-μm sterile cell strainer (Corning, NY, USA), mixed thoroughly with Percoll (Yeasen Biotech, Shanghai, China), and centrifuged at 4 °C for 10 min at 300*g*. Finally, the lower layer of cells was taken and lysed with the corresponding volume of erythrocyte lysate and inoculated into cell culture flasks, and after the cells were attached to the wall, the culture medium was replaced with DMEM [supplemented with 10% FBS, 1% penicillin/streptomycin, and 1% l-glutamine solution (Thermo Fisher, Carlsbad, CA)] for culture, and the cell viability as monitored by trypan blue (Sangon Biotech, Shanghai, China) staining could reach 90%.

### In vitro coculturing of HepG2/SynHeps-II cells with PBMCs/neutrophils

First, PBMCs/neutrophils cells were seeded in 24-well tissue culture dishes at 1 × 10^6^ cells per well and then HepG2/SynHeps-II cells were seeded at 1 × 10^6^ cells per well in the Transwell (Corning, NY, USA). After 100 μM bilirubin/3 mM NH_4_Cl was added to the 24-well plate, cells were cocultured in RPMI 1640 supplemented with 10% FBS and 1% penicillin/streptomycin. The cell culture medium was harvested for cytokine assays after 24 h of coculturing.

### Animal studies

Female C57BL/6 mice (22 to 25 g) and female New Zealand rabbits (3 to 4 kg) were used in the study. All animals were purchased from the Laboratory Animal Center of Huazhong Agricultural University (Wuhan, China). Animals were housed at a constant temperature of 25 °C and under 12-h light/dark cycle conditions with free access to food and water. All the animal protocols were reviewed and approved by the Animal Care and Use Committee (ACUC) of Huazhong Agricultural University.

ALF mouse model was established by intraperitoneal injection of 1,500 mg/kg d-galactosamine hydrochloride (d-gal; Macklin, Shanghai, China), which was dissolved in saline solution containing 0.4 mg of lipopolysaccharide (Sigma-Aldrich, St. Louis, MO) per liter. This model recapitulates the ALF profile in human. Thereafter, the mice were randomly divided into 6 groups: The first group was healthy control injected intraperitoneally with saline solution (*n* = 8 per group), the second group was the Cytopore_mouse primary hepatocytes (Cytopore_MPH, *n* = 8) treatment group, and the third to sixth groups were Cytopore_only treatment and Cytopore_cells treatment groups (Cytopore_HepG2, Cytopore_SynHeps-I, and Cytopore_SynHeps-II) (*n* = 15 per group). In the Cytopore_cells treatment group, 1 × 10^7^ Cytopore_cells were intraperitoneally injected to the liver region of the ALF mice. For the Cytopore_only and Cytopore_HepG2 treatment groups, blood and tissues were taken from mice just before the animal death for analysis. In the Cytopore_cells treatment group, blood was taken daily through the tail vein for a total of 7 days, and thereafter, mice were euthanized and tissues (liver and brain) were taken for H&E, immunohistochemical, and immunofluorescence analysis.

ALF rabbit model was established by intraperitoneal administration of d-gal (dissolved in saline solution containing 5% GLC) in healthy female animals. Pilot experiments determined the appropriate drug dose (1,000 mg/kg) and optimal treatment window. The treatment was started at 20 h after d-gal injection. Rabbits were randomized into 5 treatment groups: Cytopore_only (*n* = 6), BR350 (a nonselective bilirubin-absorbing resin, *n* = 6), Cytopore_HepG2 (*n* = 6), Cytopore_SynHeps-I (*n* = 6), and Cytopore_SynHeps-II (*n* = 10). Cytopore_cells were assembled into the PCC-BAL system for extracorporeal blood purification treatment. To monitor the process of liver damage and recovery, blood was collected from the vein at the margin of the ear every day after treatment, and the monitoring was continued for 7 days until the animals were fully recovered. For immunohistochemical and immunofluorescence analyses, some rabbits were euthanized on the 2nd (Cytopore_only group), 5th, and 7th (Cytopore_SynHeps-II group) day after d-gal induction, and paraffin sections of liver tissue were used for H&E, immunohistochemistry, and immunofluorescence analysis.

### Statistical analysis

All statistical analyses were conducted using GraphPad Prism7 (GraphPad Software, La Jolla, CA) and expressed as the mean ± SEM. Comparison of 2 groups was performed using unpaired Student’s *t* test. One-way or two-way analysis of variance (ANOVA) with Tukey’s or Bonferroni’s tests was performed when multiple groups were compared. For survival time statistics, the Mantel–Cox log-rank test was performed. All the *P* values of less than 0.05 were considered statistically significant.

## Results

### Generation of SynHeps cells

**Fig. 2.**
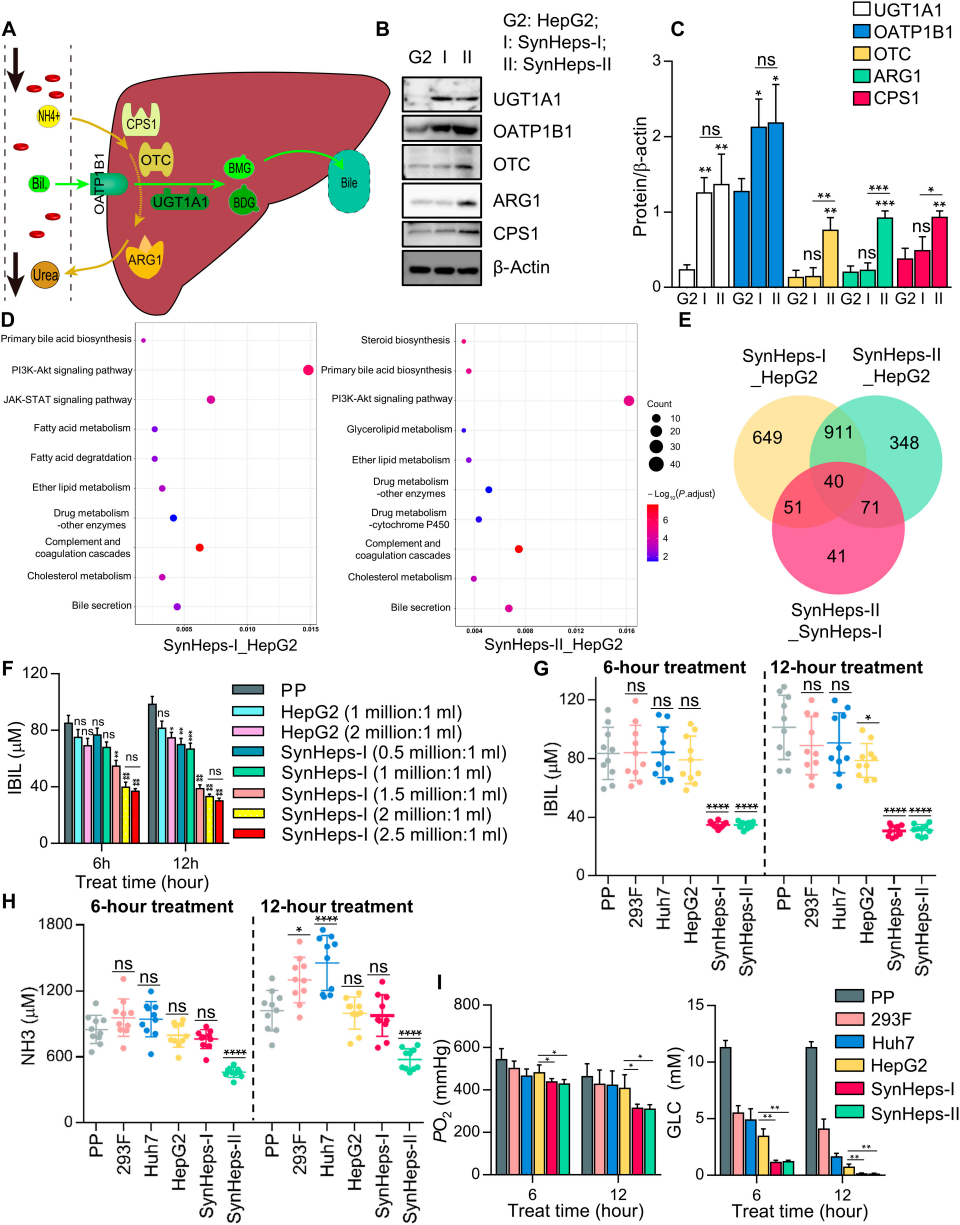
Generation of SynHeps cells. (A) Schematic illustration of bilirubin and ammonia detoxification pathway. SynHeps-I cell line was generated by overexpression of UGT1A1 and OATP1B1 in HepG2 cell line, aiming to detoxify bilirubin. SynHeps-II cell line was generated by overexpression of CPS1, OTC, and ARG1 in SynHeps-I cell, aiming to detoxify bilirubin and ammonia. (B) Western blot analysis on the expression of UGT1A1, OATP1B1, OTC, ARG1, and CPS1 in HepG2, SynHeps-I, and SynHeps-II cells. Cell lysates were separated by 10% SDS-PAGE under reducing conditions, with 50 μg of total protein per lane. (C) Quantification of band intensity from (B). *n* = 3; *P < 0.05, **P < 0.01, ***P < 0.001; ns, not significant; statistical significance was analyzed using unpaired Student’s *t* test. β-Actin was used for normalization. (D) Representative pathways by KEGG analysis that were enriched in SynHeps-I and SynHeps-II cells. HepG2 cells were used as comparison cell. (E) Venn diagram showing the number of differentially expressed genes in HepG2, SynHeps-I, and SynHeps-II. (F) Removal of unconjugated bilirubin from 80% patient plasma (PP) by different amount of HepG2 and SynHeps-I cells. Different numbers of HepG2 and SynHeps-I cells were attached to the surface of T-25 cell culture flask for 12 h, and the medium was replaced with plasma [DMEM supplemented with 80% patient plasma (PP)] from ALF patients. After 6 and 12 h of incubation, the concentration of unconjugated bilirubin (IBIL) in the plasma was measured. *n* = 3; *P < 0.05, ****P < 0.0001; statistical significance was analyzed using one-way ANOVA. (G) Comparison of different cell lines for their ability to remove bilirubin from patient plasma. The ratio of cells (in millions) to plasma (in milliliters) was fixed at 2:1. Plasma from 10 ALF patients was processed in the same way as in (F). *P < 0.05, ****P < 0.0001; statistical significance was analyzed using one-way ANOVA. (H) Comparison of different cell lines for their ability to remove ammonia from patient plasma. Experimental settings were the same as in (F). (I) Oxygen and GLC consumption data for different cell lines. *n* = 3; *P < 0.05, **P < 0.01; statistical significance was analyzed using unpaired Student’s *t* test.

Metabolism of bilirubin and ammonia is essential for the working cells in the BAL. We chose HepG2 as the parental cell line since its subclone C3A has been tested in clinical trials. Five genes were selected for expression enhancement because of their low expression levels in HepG2, namely, OATP1B1 and UGT1A1, which are involved in the hepatic uptake and conversion of toxic bilirubin, as well as CPS1, OTC, and ARG1, which are involved in the urea cycle (Fig. 2A). These genes were overexpressed in HepG2 cells by stable transfection with 2 linearized plasmids. Two cell lines were sequentially generated: SynHeps-I that was overexpressing OATP1B1 and UGT1A1, and SynHeps-II that was overexpressing all 5 genes. The elevated expression levels of the corresponding genes were confirmed by Western blot analysis (Fig. 2B and C). Transcriptome sequencing of the modified SynHeps-I and SynHeps-II with their parental HepG2 cells was performed, and the enrichment of gene expression was analyzed by Kyoto Encyclopedia of Genes and Genomes (KEGG) pathway. The results revealed significant differences between the 2 engineered cell lines in cellular proliferation pathways [e.g., phosphatidylinositol 3-kinase (PI3K)–AKT signaling pathway], followed by enhancement of cellular metabolism (e.g., drug metabolism and cholesterol metabolism), and last hepatocyte functions (e.g., bile secretion and coagulation cascade) (Fig. 2D). The overall numbers of differentially expressed genes were shown in Fig. 2E. The expression levels of functionally important or hepatocyte-specific genes were also confirmed by qPCR (Fig. S1A and B). As expected, the expression level of UGT1A1 was approximately 400-fold higher in SynHeps-I and SynHeps-II cells compared to parental HepG2 (Fig. S1A). Interestingly, the expression of some other genes such as ALB, α-1-antitrypsin (AAT), glutathione S-transferase α 1/2 (GSTA1/2), aquaporin 9 (AQP9), apolipoprotein H (APOH), epidermal growth factor receptor (EGFR), hepatocyte nuclear factor 4α (HNF-4α), and metabolizing enzymes (e.g., cytochrome P450 CYP2C8, CYP3A4, CYP4F3, CYP8B1, and CYP27A1) was also significantly increased in SynHeps-I and SynHeps-II cells (Fig. S1A and B), suggesting that these engineered cells may have gained additional improvement in other aspects.

To assess the bilirubin- and ammonia-metabolizing ability, SynHeps-I, SynHeps-II, and HepG2 cells were incubated with plasma of ALF patients for 6 and 12 h. The ratio of cells to PP [DMEM supplemented with 80% patient plasma (PP)] was titrated on SynHeps-I and HepG2 cells (Fig. 2F). A nearly maximal clearance of bilirubin was seen at 2 million SynHeps-I cells per milliliter of PP after 6 h of incubation (Fig. 2F). Concentration of unconjugated bilirubin (IBIL) in the plasma was reduced from about 80 μM to about 25 μM after incubation with SynHeps-I cells, whereas incubation with HepG2 cells only slightly reduced the concentration. We then tested and compared the bilirubin-reducing potential of the nonhepatic cell line 293F and hepatic cell line Huh7, and found that neither of them had such capacity (Fig. 2G), indicating that bilirubin metabolism was strictly dependent on the function of UGT1A1 and OATP1B1 genes in SynHeps-I and SynHeps-II cells. Similarly, due to forced overexpression of ammonia-metabolizing genes OTC, ARG1, and CPS1, only SynHeps-II cells were able to significantly reduce ammonia concentration in plasma of ALF patients from about 1,000 μM to about 450 μM (Fig. 2H). The partial pressure of oxygen (*P*o_2_) and GLC, which reflect the overall metabolic activity of the cells, were significantly lower in SynHeps-I and SynHeps-II cells than in parental HepG2 and other cell lines, especially GLC (Fig. 2I), suggesting that SynHeps-I and SynHeps-II cells acquired stronger metabolic capacity.

### Proliferation properties of SynHeps cells

**Fig. 3.**
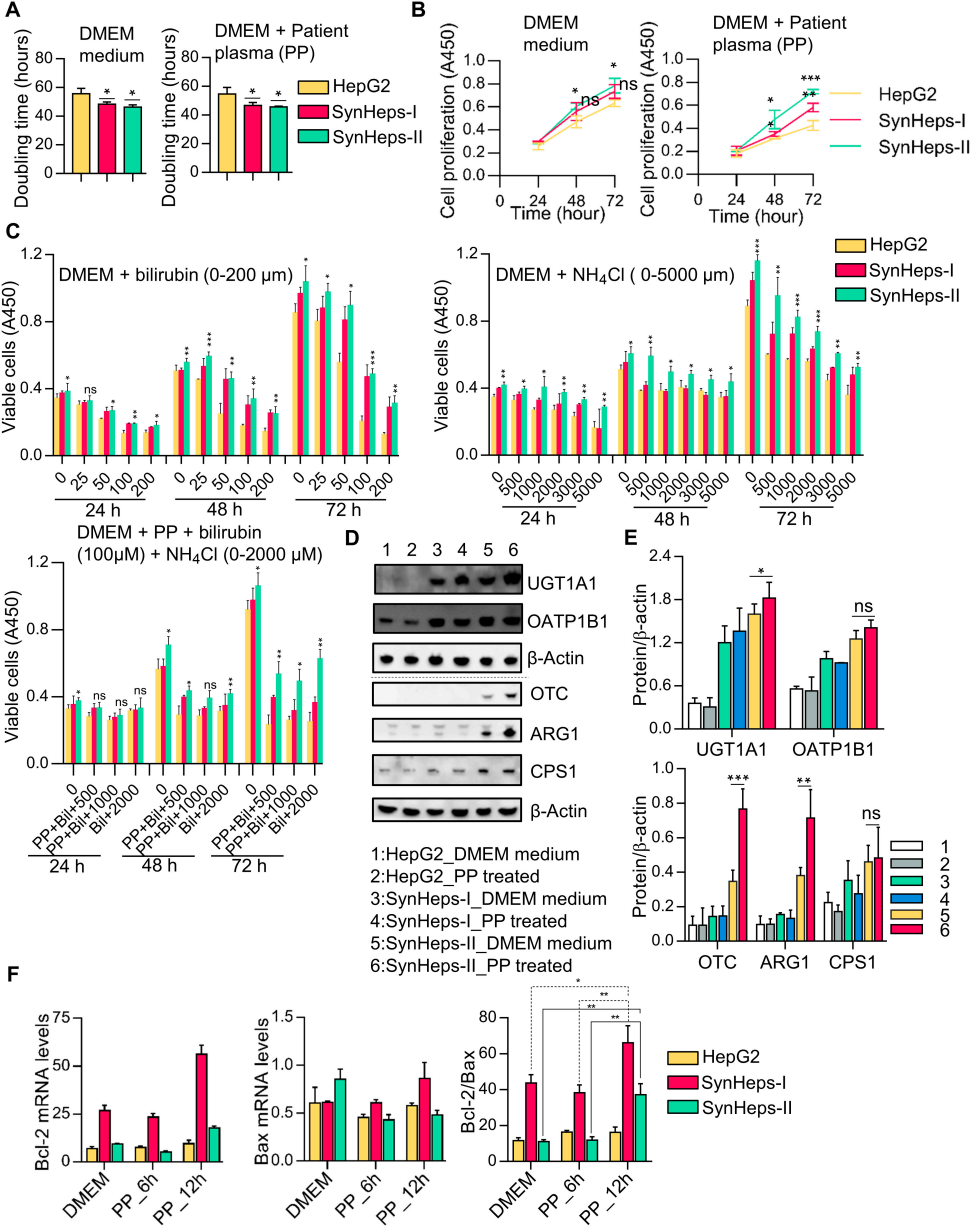
Proliferation properties of SynHeps cells. (A) Doubling time of HepG2, SynHeps-I, and SynHeps-II cells in DMEM growth medium or DMEM supplemented with 80% patient plasma (PP). The cell doubling time (DT) was calculated as DT = Δt*[lg2/(lgNt − lgNo)], where Δ*t* is the incubation time in hours, No is the number of cells counted for the first time after cell inoculation, and Nt is the cell number after Δ*t*. Cell number was counted with automated cell counter Countess 3 (Thermo Fisher, Carlsbad, CA). Each time point was counted 5 times and then averaged, and the experiment was repeated 3 times. *P < 0.05; statistical significance was analyzed using unpaired Student’s *t* test. (B) Proliferation rates of HepG2, SynHeps-I, and SynHeps-II cells in DMEM growth medium and DMEM supplemented with 80% patient plasma. Cell proliferation was measured using the CCK-8 kit following the manufacture’s protocol, and the experiment was repeated 3 times. *P < 0.05, **P < 0.01, ***P < 0.001; statistical significance was analyzed using unpaired Student’s *t* test. (C) Proliferation of HepG2, SynHeps-I, and SynHeps-II cells in DMEM supplemented with increasing concentrations of bilirubin (left) or NH_4_Cl (middle) or in a mixture of 80% patient plasma (PP) supplemented with 100 μM bilirubin and increasing concentrations of NH_4_Cl (right). Cell proliferation was measured with a CCK-8 kit and expressed as A450 values determined by multimode microplate reader. Statistical analyses were based on comparisons between SynHeps-II and HepG2 at the same time period and processing conditions. *n* = 3; *P < 0.05, **P < 0.01, ***P < 0.001; statistical significance was analyzed using unpaired Student’s *t* test. (D) Western blot analysis on the expression of UGT1A1, OATP1B1, OTC, ARG1, and CPS1 in HepG2, SynHeps-I, and SynHeps-II cells. Cells were grown in DMEM or DMEM supplemented with 80% PP for 24 h prior to the assay (*n* = 3). (E) Quantification of band intensity in (D). β-Actin was used for normalization. *P < 0.05, **P < 0.01, ***P < 0.001; statistical significance was analyzed using unpaired Student’s *t* test. (F) Expression of Bax and Bcl-2 genes in HepG2, SynHeps-I, and SynHeps-II cells cultured in DMEM or DMEM supplemented with 80% patient plasma (PP) for 6 and 12 h (*n* = 3). *P < 0.05, **P < 0.01; statistical significance was analyzed using unpaired Student’s *t* test. The assay was performed by qPCR. Bcl-2/Bax was the ratio of Bcl-2 to Bax.

The proliferative properties of the working cells in the BAL impact the manufacturing process and cost. It was found that the engineered SynHeps-I and SynHeps-II cells had excellent expansion properties, especially the latter. As shown in Fig. 3A, the doubling time of SynHeps-I and SynHeps-II cells was significantly shorter compared to parental HepG2 cells, both in DMEM growth medium and in DMEM supplemented with 80% patient plasma (PP). For consistency, the cell proliferation rates of both SynHeps-I and SynHeps-II were increased, especially for SynHeps-II cells (Fig. 3B). Since high concentrations of serum bilirubin and ammonia from ALF patients are toxic to hepatocytes, to understand the tolerance of SynHeps cells to these unfavorable conditions, we cultured the cells in DMEM supplemented with either 0 to 200 μM bilirubin, or 0 to 5,000 μM NH_4_Cl, or 80% of the patient plasma mixed with 100 μM bilirubin and 0 to 2,000 μM NH_4_Cl to simulate the most challenging conditions (Fig. 3C). The results showed that SynHeps-II cells had a significantly better proliferative capacity than HepG2 cells and a much better tolerance to high concentrations of bilirubin (up to 200 μM) and ammonia (up to 5,000 μM) than HepG2 cells under all tested conditions. After culturing the cells in 80% patient plasma for 24 h, we examined the expression of bilirubin- and ammonia-metabolizing genes UGT1A1, OATP1B1, OTC, ARG1, and CPS1 by Western blotting (Fig. 3D and E). The results showed that coincubation with patient plasma did not negatively affect the expression of all these genes. In contrast, exposure to patient plasma significantly up-regulated the expression of UGT1A1, OTC, and ARG1 genes in SynHeps-II cells. Similarly, the expression of proapoptotic marker Bax and anti-apoptotic marker Bcl-2 in HepG2, SynHeps-I, and SynHeps-II cells was analyzed by qPCR (Fig. 3F), which showed a significant increase in the Bcl-2/Bax ratio in SynHeps-I and SynHeps-II cells, but not in HepG2 cells, after 6 and 12 h of exposure to the 80% patient plasma, suggesting that the engineering of the bilirubin pathway increased the cell viability.

### 3D culturing of SynHeps cells in microcarrier Cytopore

**Fig. 4.**
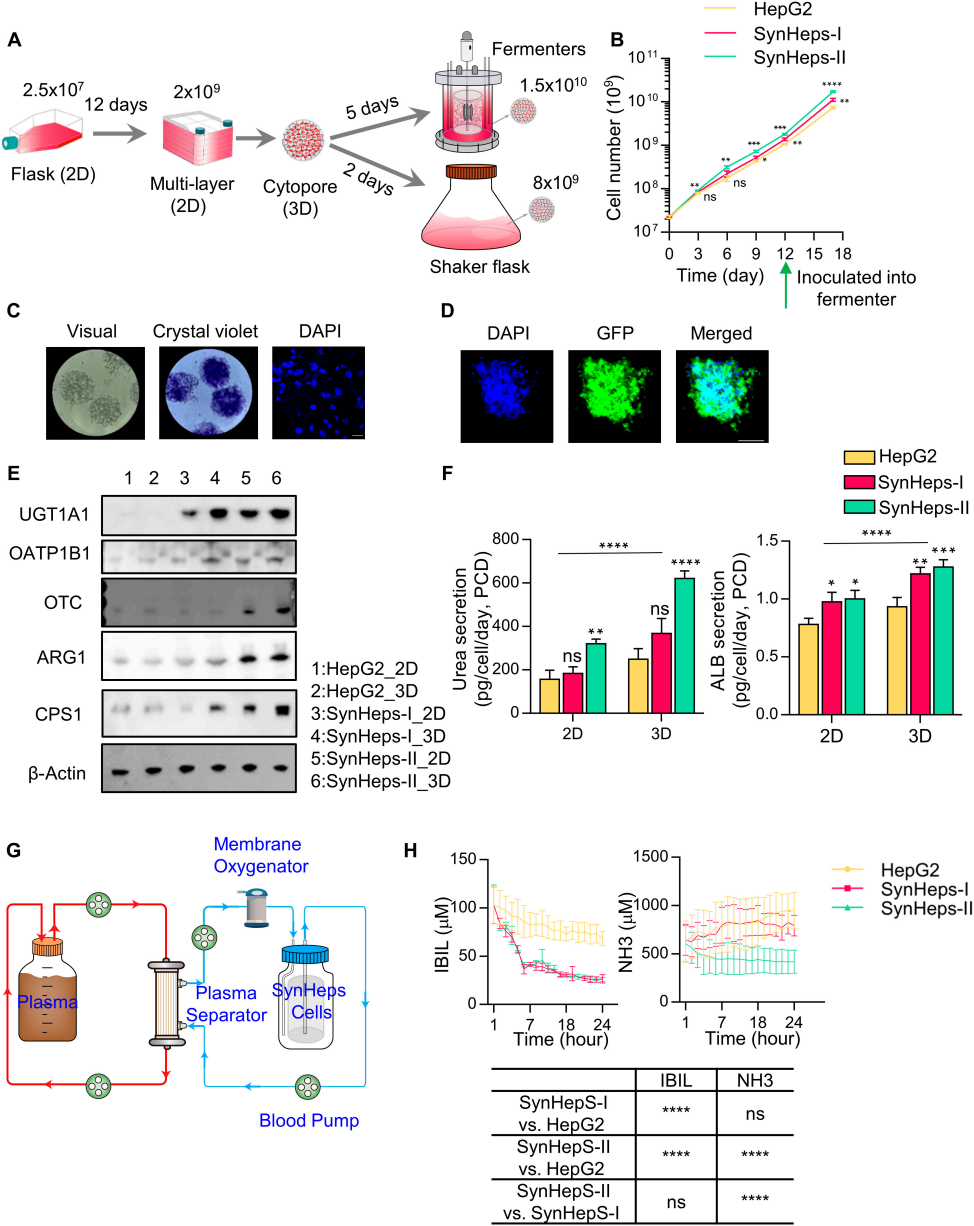
3D expansion of hepatocytes in Cytopore. (A) Schematic diagram of large-scale cell expansion strategy. Cells were initially grown in cell culture flasks (T-175) and then expanded in a multilayer cell culture system to approximately 2 × 10^9^. Thereafter, cells were transferred to shake flasks or fermenter for further expansion. (B) Expansion curves of HepG2, SynHeps-I, and SynHeps-II cells from approximately 2.5 × 10^7^ to approximately 1.5 × 10^10^ cells within 17-day period (*n* = 3). *P < 0.05, **P < 0.01, ***P < 0.001, ****P < 0.0001; statistical significance was analyzed using unpaired Student’s *t* test. (C) Microscope images of SynHeps-II cells cultured on Cytopore microcarrier (left) and stained with 0.1% crystal violet (middle) or DAPI (right). Scale bar, 25 μm. (D) Fluorescence imaging of SynHeps-II cells transfected with GFP coding gene, grown in Cytopore, and stained with DAPI (blue, left) or imaged by endogenous fluorescence of GFP (green, middle). Scale bar, 100 μm. (E) Protein expression levels of UGT1A1, OATP1B1, OTC, ARG1, and CPS1 determined by Western blot after 3 days of plate culturing (2D) and microcarrier culturing (3D). (F) Synthesis of ALB and urea in HepG2, SynHeps-I, and SynHeps-II cells after 3 days of plate culturing (2D) and microcarrier culturing (3D). *n* = 3; *P < 0.05, **P < 0.01, ***P < 0.001; statistical significance was analyzed by two-way ANOVA. (G) Schematic illustration of extracorporeal blood purification method using ALF patient plasma. The diagram showed the plasma separator (OP-08), membrane oxygenator, micro-BAL (loaded hepatocytes), and blood pump. (H) Continuous measurement of unconjugated bilirubin [indirect bilirubin (IBIL)] and NH3 during the extracorporeal blood purification process (*n* = 3 per group). ****P < 0.0001; statistical significance was analyzed by one-way ANOVA.

In order to avoid mechanical damage to the hepatocyte caused by the agitation and flow of the plasma during cell production and working period, we established a 3D cell culturing system by growing cells in the spherical microcarrier Cytopore (Fig. 4A and Fig. S2). First, the stock cells were thawed and cultured in T-175 cell culture flasks at 2.5 × 10^7^ cells and expanded to 6 × 10^8^ cells, and then cultured in the stationary multilayer cell manufacturing system for 3 days, typically yielding 2 × 10^9^ cells. These cells were detached and mixed with Cytopore and cultured in a fermenter for additional 5 days, typically yielding 1.5 × 10^10^ cells. Alternatively, the cells were detached and mixed with Cytopore and cultured in a shaker flask for 2 days, typically yielding 8 × 10^9^ cells. The final collection of Cytopore_cell complexes was used for functional testing or loaded into a BAL bioreactor for in vitro blood purification therapy using patient plasma or the ALF rabbit model. The cells grew well in the fermenter and expanded rapidly and exponentially (Fig. 4B). Staining of Cytopore_cell spheres with crystal violet and DAPI showed that the cells in the spheres were evenly distributed and dispersed (Fig. 4C). This was further confirmed by culturing GFP-expressing SynHeps-II cells in Cytopore and observing the cells under a fluorescence microscope (Fig. 4D). In terms of gene expression, 3D-expanded cells showed higher expression levels of UGT1A1, OTC, ARG1, and CPS1 compared to 2D-cultured cells (Fig. 4E). Further, 3D-cultured cells formed more urea and secreted more ALB compared to 2D-cultured cells (Fig. 4F). Based on the 3D-cultured SynHeps cells, a prototype of the BAL system (named PCC-BAL) was built for the simulated extracorporeal blood purification experiment using ALF patient plasma. The system included the Cytopore_cell complexes, membrane plasma separator (OP-08), membrane oxygenator, and blood pumps (Fig. 4G). A collection of 1.2 × 10^10^ Cytopore_cells was tanked into the PCC-BAL bioreactor for the purification of 2.5 l of patient plasma. The plasma was circulated throughout the bioreactor at a flow rate of 50 ml/min, while oxygen and GLC were supplemented throughout the process. The purification process lasted 24 h, and plasma samples were taken for analysis at set time points. As expected, with treatment by SynHeps-II-loaded PCC-BAL, plasma concentrations of unconjugated bilirubin (IBIL) and ammonia decreased progressively over time (Fig. 4H), whereas SynHeps-I could only reduce bilirubin concentrations (Fig. 4H).

### Treatment of ALF mouse model with Cytopore_SynHeps cells

**Fig. 5.**
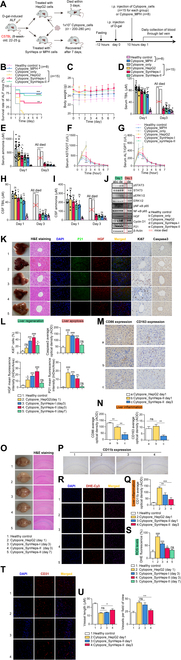
Treatment of ALF mouse model with Cytopore_cells. (A) Schematic diagram of animal experimental design. ALF mice were induced by intraperitoneal injection of d-gal, and if left untreated, 90% of the mice died spontaneously within 48 h. Ten hours later after d-gal injection, mice were treated by intraperitoneal injection of Cytopore_cells to the liver region and Cytopore_only as cell carrier control. Blood samples were daily collected for metabolite analysis. (B) Survival curves of healthy mice, and ALF mice treated with Cytopore_only, Cytopore_cells (*n* = 15 per group), and Cytopore_mouse primary hepatocytes (MPH) (*n* = 8). **P < 0.01, ***P < 0.001, ****P < 0.0001; statistical significance was analyzed by log-rank (Mantel–Cox) test. (C) Body weight of healthy mice, and ALF mice treated with Cytopore_cells or Cytopore_only. (D) Serum total bilirubin (TBIL) concentrations in healthy mice or ALF mice treated with Cytopore_only, Cytopore_cells, and Cytopore_MPH. Day 3 data in the Cytopore_only and Cytopore_HepG2 groups were not available because all mice in these groups died within 70 h of d-gal induction. (E) Serum ammonia concentrations in healthy mice or ALF mice treated with Cytopore_only, Cytopore_cells, and Cytopore_MPH. (F) Serum levels of aspartate aminotransferase (AST) in healthy mice or treated ALF mice over a 7-day period. (G) Serum levels of alanine aminotransferase (ALT) in healthy mice or treated ALF mice over a 7-day period. All data in (F) and (G) were statistically analyzed on day 2 post-treatment, and Cytopore_SynHeps-II was compared to Cytopore_only. ****P < 0.0001; statistical significance was analyzed using unpaired Student’s *t* test. (H) TBIL concentrations in cerebrospinal fluid (CSF) of healthy mice or ALF mice treated with Cytopore_only, Cytopore_cells, and Cytopore_MPH. (I) Ammonia concentrations in CSF of healthy mice or ALF mice treated with Cytopore_only, Cytopore_cells, and Cytopore_MPH. Statistical significance of (D), (E), (H), and (I) was analyzed using unpaired Student’s *t* test. *P < 0.05, **P < 0.01, ***P < 0.001, ****P < 0.0001. (J) Western blot analysis on the expression level of signaling proteins in liver regeneration, apoptosis, and inflammation from the livers of healthy mice and mice treated with Cytopore_only and Cytopore_HepG2 (day 1 after d-gal induction) and Cytopore_SynHeps-I and SynHeps-II (days 1 and 3 after d-gal induction). (K) Morphology, H&E, immunofluorescence, and immunohistochemical staining of liver sections from mice treated with Cytopore_HepG2, Cytopore_SynHeps-I, and Cytopore_SynHeps-II cells to visualize the expression of P21, HGF, Ki67, and Caspase3. Untreated healthy mice were used as control. Anti-HGF antibody was labeled with Cy3, and anti-P21 antibody was labeled with Alexa Fluor 488. Images were taken using a confocal microscope. Scale bar, 25 μm. (L) Quantification of liver regeneration markers Ki67 and HGF, and liver apoptosis markers P21 and Caspase3 from (K). *n* = 3; *P < 0.05, **P < 0.01, ***P < 0.001, ****P < 0.0001; statistical significance was analyzed by one-way ANOVA. (M) Immunohistochemical staining of the liver sections from mice treated with Cytopore_HepG2 (day 1 after d-gal induction) and Cytopore_SynHeps-II cells (days 1 and 3 after d-gal induction) to visualize the expression of CD86 and CD163. Scale bar, 25 μm. (N) Quantification of liver inflammation markers CD86 and CD163 from (M). *n* = 3; *P < 0.05, **P < 0.01, ****P < 0.0001; statistical significance was analyzed by one-way ANOVA. (O) Morphology and H&E staining of brain tissue sections from healthy mice or mice treated with Cytopore_HepG2, Cytopore_SynHeps-I, and Cytopore_SynHeps-II cells. Scale bar, 100 μm. (P) Immunohistochemical staining of the brain tissue sections from healthy mice or mice treated with Cytopore_HepG2 (day 1 after d-gal induction) and Cytopore_SynHeps-II cells (days 1 and 3 after d-gal induction) to visualize the expression of CD11b. Scale bar, 100 μm. (Q) Quantification of CD11b expression from (P). *n* = 3; **P < 0.01, ***P < 0.001; statistical significance was analyzed by one-way ANOVA. (R) Hippocampal ROS assay in mouse brain at different time points after treatment with Cytopore_HepG2 and Cytopore_SynHeps cells and in brain of healthy mice. The fluorescent probe was DHE-Cy3-labeled and used in the assay. Scale bar, 50 μm. (S) Quantitative analysis of ROS levels from (R). *n* = 3; *P < 0.05, ***P < 0.001, ****P < 0.0001; statistical significance was analyzed by one-way ANOVA. (T) Expression of CD31 in the brain tissue of mice treated with Cytopore_HepG2 (day 1 after d-gal induction) and Cytopore_SynHeps-II (days 1 and 3 after d-gal induction) and in brain tissues of healthy mice. Scale bar, 25 μm. (U) Quantification of brain vessel length and density, which was calculated from (T). *n* = 3; *P < 0.05, **P < 0.01; statistical significance was analyzed by one-way ANOVA.

To establish an ALF mouse model, C57 mice were intraperitoneally injected with 1,500 mg/kg d-gal (dissolved in saline solution containing 0.4 mg of lipopolysaccharide per liter), and these mice developed signs of fulminant hepatic failure within 48 h, with a mortality rate of up to 90%. Ten hours after d-gal induction, treatment was started by intraperitoneal injection of Cytopore_cell complexes to the liver region (Fig. 5A). Mice in the healthy control group received PBS solvent and remained alive, while in the Cytopore_only treatment group, all mice died within 48 h, whereas Cytopore_HepG2-treated mice died within 48 to 68 h. In the groups treated with Cytopore_SynHeps-I, Cytopore_SynHeps-II, and Cytopore_MPH, 40% (6 of 15, *P* = 0.024), 66.7% (10 of 15, *P* < 0.0001), and 75% (6 of 8, *P* = 0.0003) of mice survived, respectively (Fig. 5B). During ALF period, mice had reduced food intake and significant weight loss. After treatment, mice gradually became active and their body weight began to increase to the normal range (Fig. 5C). No other obvious side effects were observed in the treated mice. In the Cytopore_SynHeps- and Cytopore_MPH-treated group, serum levels of TBIL (Fig. 5D), ammonia (Fig. 5E), AST (Fig. 5F), and ALT (Fig. 5G) declined from peak levels on days 1 to 2 after d-gal induction to baseline levels on day 3, whereas levels of these biomarkers in the Cytopore_only or Cytopore_HepG2-treated group remained elevated, and all mice died eventually. The TBIL and ammonia levels in the CSF showed a trend similar as in the serum (Fig. 5H and I). Finally, in order to confirm the advantages and necessity of the 3D-cultured Cytopore_SynHeps-II cell therapy [30], we compared the therapeutic effects of Cytopore_SynHeps-II and SynHeps-II alone on ALF mice, and we found that the survival rate of the mice in the Cytopore_SynHeps-II treatment group was significantly higher than in the SynHeps-II_only treatment group, and at the same time, the prognostic indexes such as serum TBIL, ammonia, AST, and ALT were also more normalized (Fig. S3A).

Previous studies indicated that high concentrations of bilirubin could promote hepatic inflammation through activation of NF-κB pathway [8]. To explore this possibility, we performed Western blot analysis of liver tissues from treated mice, which showed that the expression of liver regeneration-related markers pSTAT3, pERK1/2, HGF, and Cyclin D1 was significantly up-regulated on day 1 after Cytopore_SynHeps-I and Cytopore_SynHeps-II treatment compared with Cytopore_only and Cytopore_HepG2 treatment, whereas the expression of liver inflammation and cell death-related markers pNF-κB and P21 was significantly down-regulated (Fig. 5J and Fig. S3B and C). Similarly, immunohistochemical and immunofluorescence analyses showed that Cytopore_SynHeps treatment ameliorated liver injury and promoted liver regeneration. Compared with Cytopore_HepG2 treatment, Cytopore_SynHeps-I and Cytopore_SynHeps-II treatment significantly up-regulated the expression of Ki67 and HGF, markers related to cell proliferation and liver regeneration, and down-regulated the expression of P21 and Caspase3, markers related to liver injury (Fig. 5K and L). On day 7 after Cytopore_SynHeps-II treatment, the liver almost returned to the normal state of the healthy control group without obvious bleeding or liver lesions (Fig. 5K, 5th panel). Since Kupffer cells are the main innate immune cells in the liver, and CD86 and CD163 are the activation and polarization markers for macrophages, respectively, we explored the inflammatory state of the liver by immunohistochemical staining of CD86 and CD163. As shown in Fig. 5M and N, the expression of CD86 on day 1 after Cytopore_HepG2 treatment was much higher than that after Cytopore_SynHeps-II treatment, indicating that Cytopore_SynHeps-II treatment ameliorated the inflammatory state of the liver. On day 3 after Cytopore_SynHeps-II treatment, CD86 and CD163 expression was further down-regulated. The serum levels of interleukin-1β (IL-1β), IL-6, and TNF-α were also significantly higher in the Cytopore_only and Cytopore_HepG2 treatment groups than in the Cytopore_SynHeps-I, Cytopore_SynHeps-II, and Cytopore_MPH treatment groups (Fig. S3D). In conclusion, these data suggested that the liver in ALF was in a state of severe inflammation, and Cytopore_SynHeps-I and Cytopore_SynHeps-II treatments significantly improved the condition, especially the latter.

In addition to liver injury, d-gal-induced ALF mice also developed hepatic encephalopathy, which manifested as anorexia, unsteady movement, inactivity, and delayed response to external stimuli (Movie S1). As mentioned above, the concentrations of bilirubin and ammonia in the CSF of the ALF mice were markedly elevated (Fig. 5H and I). Mice presenting with ammonia encephalopathy showed mildly enlarged brains (Fig. 5O, 2nd panel). H&E staining analysis showed that the Cytopore_HepG2 group had remarkable inflammation near the hippocampus of the mice, with the presence of massive cellular necrosis, structural abnormality of the brain tissue, loosening of the striated structure of the tissue, and the presence of sparing edema in the neuronal cells, and some of them were edematous to the point of vacuolated degeneration. After 3 days of treatment with Cytopore_SynHeps (Fig. 5O, 3rd and 4th panels), the swelling phenomenon of the brain shape has been improved. H&E staining analysis showed that the overall structural arrangement of the brain tissue in the overall structure of the regularity of the presence of a small number of neurons consolidation necrosis, the hippocampus near the hippocampus did not have obvious inflammatory cell infiltration. After treatment with Cytopore_SynHeps-II for 7 days (Fig. 5O, 5th panel), the shape of the brain was basically the same as that of healthy mice, and H&E staining also showed that the structure of the brain tissue was well aligned, and inflammation and necrosis were resolved. This was consistent with the reduced immunohistochemical staining of CD11b, which was the activation marker of neutrophils and macrophages, in the brain tissue sections (Fig. 5P and Q). Moreover, we also stained some brain tissue sections with DHE-Cy3 and found that the ROS level was related to the recovery time of the mice brain. The highest ROS level was detected on the first day in the mice that were not successfully treated with Cytopore_HepG2, and the ROS level was significantly reduced after treatment with Cytopore_SynHeps, and by the end of the 7 days after treatment with Cytopore_SynHeps-II, the ROS was basically restored to the base level of the normal mice (Fig. 5R and S). Interestingly, we found that high levels of ammonia and bilirubin back into the brain can penetrate the blood–brain barrier of brain tissue and appear to affect the continuity and density of blood vessels. In the brain tissue of mice treated with Cytopore_HepG2, we found that there were partial breaks in the blood vessels and an increased proportion of short blood vessels, whereas treatment with Cytopore_SynHeps-II alleviated the broken blood vessels and showed their continuity as the treatment time was prolonged (Fig. 5T and U).

Taken together, SynHeps-II cells with enhanced bilirubin and ammonia metabolism not only scavenged the toxic metabolites but also attenuated the inflammatory state in the liver and brain. Compared with SynHeps-I cells, SynHeps-II cells more effectively rescued ALF mice and alleviated the development of ammonia encephalopathy.

### Treatment of ALF rabbit model with PCC-BAL

The ALF rabbit model was established by intraperitoneal injection of 1,000 mg/kg d-gal (dissolved in saline solution containing 5% GLC) into New Zealand rabbits. After injection, rabbits developed obvious ALF symptoms such as significant elevation of serum ALT, AST, ammonia, and TBIL within 20 h. Furthermore, rabbits showed signs of yellowing of urine, loss of appetite, depression, and unsteadiness of standing (Movie S2). Based on the appearance of these symptoms, treatment was started at 20 h after d-gal injection.

**Fig. 6.**
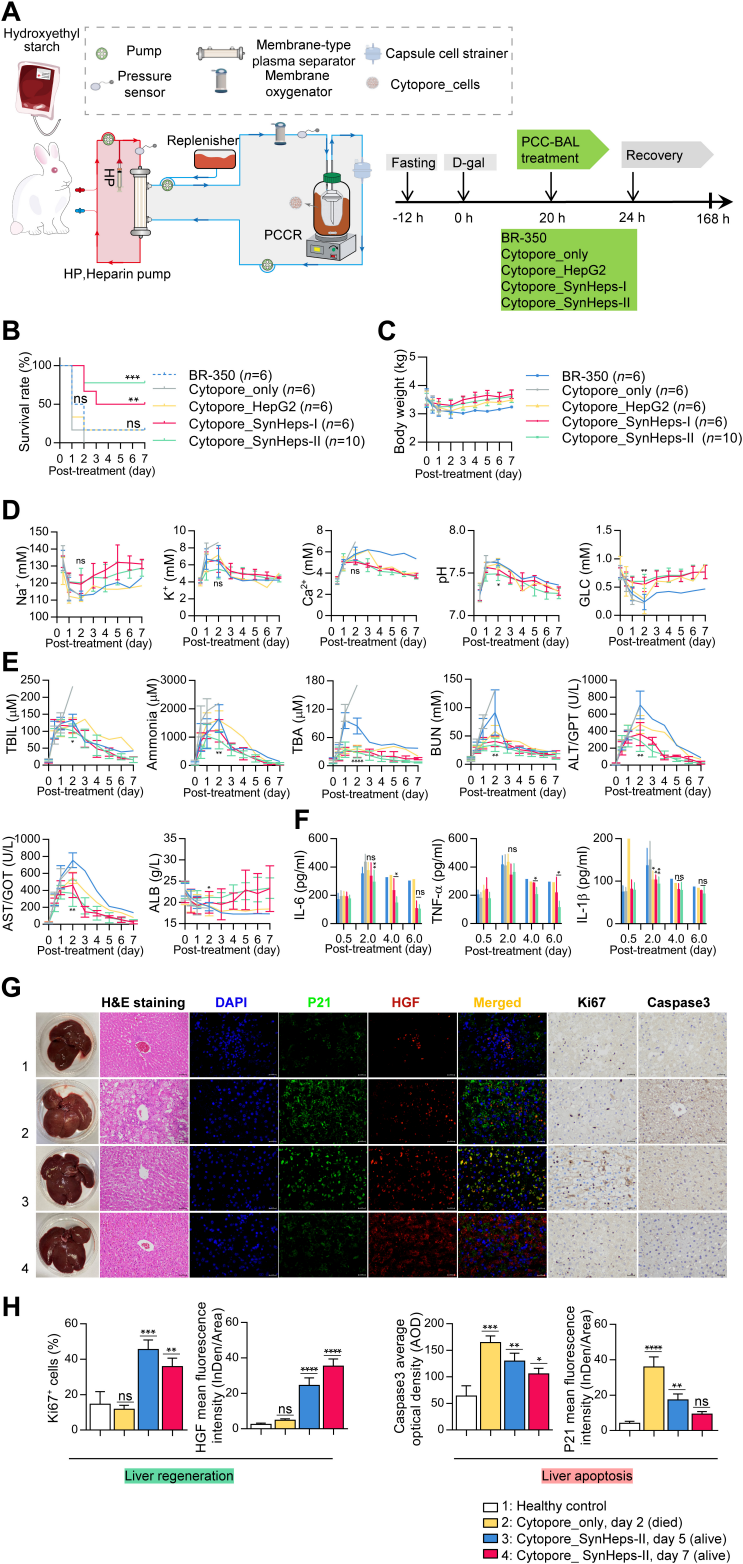
Treatment of ALF rabbit model with PCC-BAL. (A) Schematic illustration of the PCC-BAL system, which consisted of 2 parts, where the entire blood circulation part was represented by a red background schematic on the left, with red arrows indicating the direction of blood flow at a rate of 24 ml/min, and the rest of the plasma circulation part was represented by a gray background schematic on the right, with blue arrows indicating the direction of plasma flow at a rate of 8 ml/min. The whole system had 3 micro-pumps, a heparin pump (HP), a pressure sensor, a membrane-type plasma separator, a replenisher, a membrane oxygenator, a PCCR loaded with Cytopore_cells, a capsule cell strainer that prevented the Cytopore_cells from leaking and entering the circulation, and a stirring device that could warm the circulating plasma. The volume of the entire circulation was approximately 70 ml. (B) Survival curves of the rabbits treated with BR350 (a bilirubin-absorbing cartridge), Cytopore_only, Cytopore_HepG2, Cytopore_SynHeps-I, and Cytopore_SynHeps-II BAL. **P < 0.01, ***P < 0.001; statistical significance was analyzed by log-rank (Mantel–Cox) test. (C) Body weight of the treated rabbits. (D) Blood gas testing of Na^+^, K^+^, Ca^2+^, serum pH, and GLC. (E) Dynamics of blood concentrations of TBIL, ammonia, total bile acid (TBA), blood urea nitrogen (BUN), ALT, AST, and albumin (ALB). All data in (D) and (E) were Cytopore_SynHeps-II with BR350 statistically analyzed on day 2 after treatment by unpaired Student’s *t* test. *P < 0.05, **P < 0.01, ****P < 0.0001. (F) Blood concentrations of IL-6, TNF-α, and IL-1β from the treated rabbits. Statistical analyses were performed in the Cytopore_cells group versus BR350 on day 2 after treatment, and Cytopore_SynHeps-II versus Cytopore_SynHeps-I on days 4 and 6 after treatment. *P < 0.05, **P < 0.01; statistical significance was analyzed using unpaired Student’s *t* test. (G) Morphology, H&E, immunohistochemical, and immunofluorescence staining of liver sections from rabbits treated with Cytopore_only and Cytopore_SynHeps-II cells to visualize the expression of P21, HGF, Ki67, and Caspase3. Untreated healthy rabbits were used as control. Anti-HGF antibody was labeled with Cy3, and anti-P21 antibody was labeled with Alexa Fluor 488. Images were taken using a confocal microscope. For H&E images, scale bar was 50 μm, and for immunohistochemistry and immunofluorescence images, scale bar was 25 μm. (H) Quantification of liver regeneration markers Ki67 and HGF, and liver apoptosis markers P21 and Caspase3 from (G). *n* = 3; *P < 0.05, **P < 0.01, ***P < 0.001, ****P < 0.0001; statistical significance was analyzed by one-way ANOVA.

A specially designed PCC-BAL was used for the therapy via extracorporeal blood purification method (Fig. 6A). The PCC-BAL consisted of the blood circulation part and plasma circulation part. The blood circulation part was composed of a micro-pump, a heparin pump, and a membrane-type plasma separator. It pumped the blood out of the body and returned the plasma separator-filtered blood and purified plasma back into the body. The plasma circulation part consisted of 2 micro-pumps, a replenisher (rehydration device for replenishing body fluids before, during, and after treatment), a membrane oxygenator, a plasma-cell contacting reactor (PCCR) that was filled with the hepatocytes, a capsule cell strainer that prevented the cells from leaking and draining into the body, and a magnetic stirrer with warming function that mixed the Cytopore_cells with plasma to avoid cell sedimentation. Blood exited the body via carotid artery catheter, flowed through the plasma filter, and entered the collateral plasma circulation. After passing through the membrane oxygenator, the oxygen-enriched plasma flowed through the PCCR and then re-entered the capsule cell strainer. Finally, the purified plasma merged with the plasma separator-filtered blood and flowed back into the body through a cutaneous vein catheter (Movie S3).

A total of 34 adult New Zealand rabbits were randomly assigned to 5 groups, each of which either received PCC-BAL treatment loaded with different cells (HepG2, SynHeps-I, or SynHeps-II), or Cytopore_only treatment, or with BR350 (a nonselective bilirubin-absorbing resin loaded into a similar cartridge) treatment. The entire treatment lasted 3 h. As shown in Fig. 6B, treatment with SynHeps-II BAL resulted in 80% of the rabbits (8 of 10) surviving to the endpoint (day 7), while SynHeps-I BAL also showed a good efficacy, with 50% of the rabbits (3 of 6) having survived. Cytopore_only did not save any of the rabbits (*n* = 6), whereas BR350 and HepG2 BAL saved 16.7% of the rabbits (1 of 6). During the whole treatment process, the animals breathed smoothly, and no other complications were observed, and their body weight remained relatively stable after treatment (Fig. 6C). Blood gas testing showed that the blood indices such as concentrations of Na^+^, K^+^, Ca^2+^, pH value, and GLC remained stable and within the normal range (Fig. 6D), indicating that the PCC-BAL system has excellent safety profile and efficacy for the treatment of ALF, which was also reflected by the good prognosis of rabbits (Movie S4). SynHeps-I and SynHeps-II treatment significantly reduced serum levels of bilirubin, ammonia, TBA, and BUN, from the peak levels around day 2 to very low levels after day 5 (Fig. 6E). As important indicators of liver injury, the levels of ALT and AST also dropped from the peak levels at around day 2 to extremely low levels after day 5 (Fig. 6E). Serum ALB, which indicates the synthetic capacity of the liver, was significantly decreased before treatment and gradually returned to the normal range after SynHeps-I and SynHeps-II treatment, but remained low in the BR350 and HepG2 control groups (Fig. 6E). Serum levels of proinflammatory cytokine IL-6 and TNF-α were significantly reduced in the SynHeps-I and SynHeps-II treatment groups, especially the latter, compared to HepG2 and BR350 control (Fig. 6F), suggesting that SynHeps-I and SynHeps-II treatments ameliorated the inflammatory state of the animals.

Anatomical examination revealed that the peritoneal surface of the liver in the Cytopore_only group was rough, with visible foci and numerous hemorrhagic spots, and the liver morphology began to collapse (Fig. 6G, 1st column). H&E staining of the liver sections showed a large number of necrotic hepatocytes (surrounded by apoptotic vesicles), discrete and vacuolated internal structures, and inflammatory infiltration (Fig. 6G, 2nd panel). Immunohistochemical staining showed that the expression levels of HGF and Ki67, protein markers that promote liver regeneration and indicate hepatocyte proliferation, respectively, were significantly higher in the SynHeps-II treatment group than in the Cytopore_only control (Fig. 6G and H). The expression levels of P21 and Caspase3, protein markers that promote hepatocyte death and apoptosis, were much lower in the SynHeps-II treatment group than in the Cytopore_only control (Fig. 6G and H). In the SynHeps-II treatment group, the expression level of HGF on day 7 was higher than that on day 5, while the expression levels of P21 and Caspase3 on day 7 were lower than those on day 5. The expression of HGF exceeded that of P21 and Caspase3 on day 7 (Fig. 6G), indicating that the injured liver was in a post-regenerative state.

To summarize, the current PCC-BAL was able to efficiently metabolize toxic bilirubin and ammonia, ameliorate hepatocyte apoptosis and necrosis, suppress the inflammatory state, and ultimately promote liver regeneration.

### High concentration of bilirubin induced apoptosis in hepatocyte and neuron cells, and activated NF-κB pathway in immune cells

**Fig. 7.**
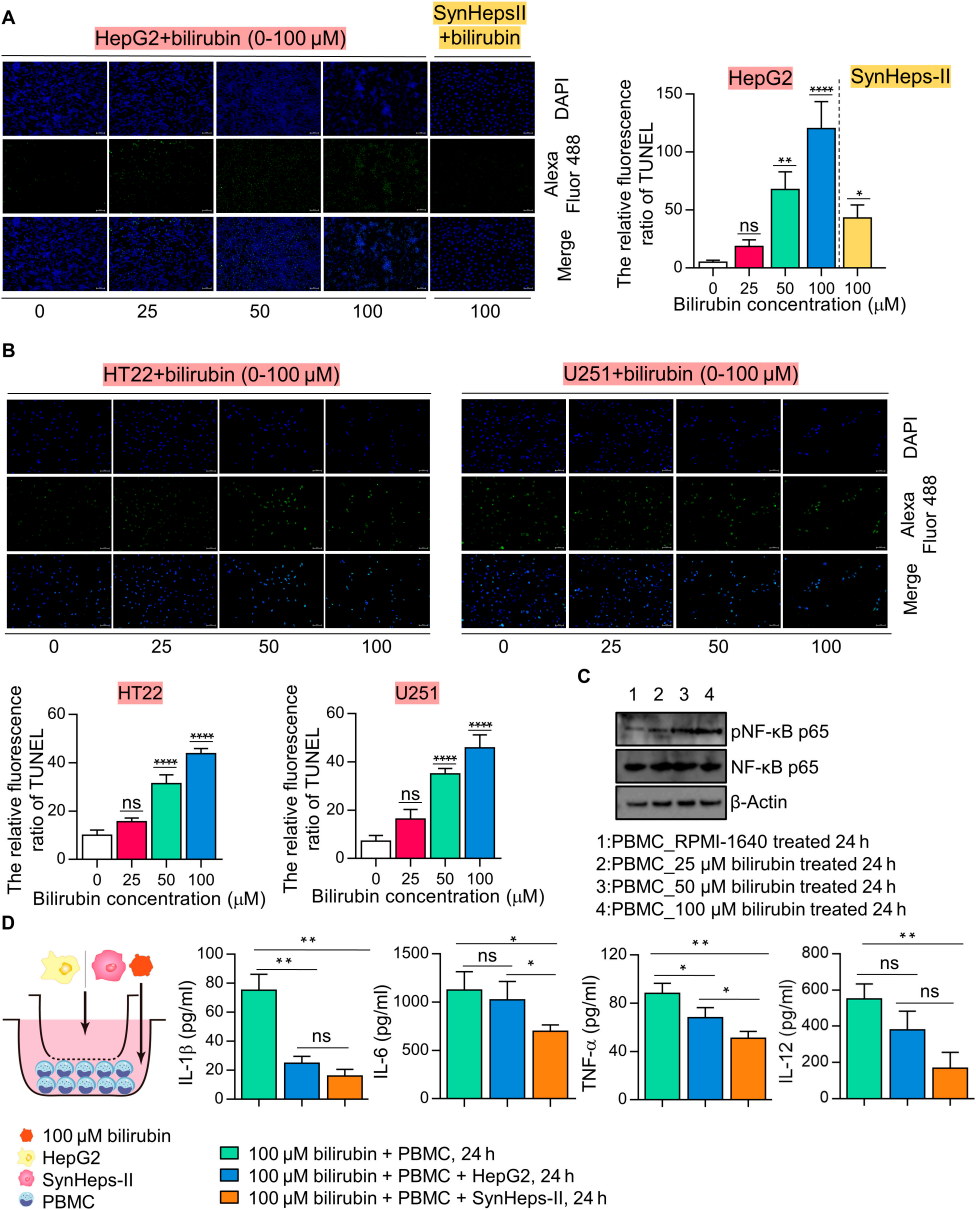
High concentration of bilirubin induced apoptosis in hepatocyte and neuron cells, and activated NF-κB pathway in immune cells. (A) High concentration of bilirubin induced hepatocyte apoptosis. HepG2 and SynHeps-II cells were incubated with increasing concentrations of bilirubin for 24 h. Cells were stained with fluorescent dye DAPI and TUNEL kit. Scale bar, 50 μm. Quantification of fluorescence intensity was shown on the right. *n* = 3; *P < 0.05, **P < 0.01, ****P < 0.0001; statistical significance was analyzed by one-way ANOVA. (B) High concentration of bilirubin induced neuronal cells (HT22) and astrocytes (U251) apoptosis. HT22 and U251 cells were incubated with increasing concentrations of bilirubin for 24 h and stained with fluorescent dye DAPI and TUNEL probe kit. Scale bar, 50 μm. Quantification of fluorescence intensity was shown below. *n* = 3; ****P < 0.0001; statistical significance was analyzed by one-way ANOVA. (C) Western blot analysis of NF-κB signaling molecules NF-κB p65 and phosphorylated NF-κB p65. PBMCs were incubated with increasing concentrations of bilirubin for 24 h. Cell lysate was prepared, separated on SDS-PAGE, and probed with corresponding antibodies. (D) PBMCs were seeded in 24-well tissue culture plate at 1 × 10^6^ cells per well, and then HepG2/SynHeps-II cells were seeded at 1 × 10^6^ cells per well in the Transwell. Then, 100 μM bilirubin was added to the 24-well plate, and cells were cocultured in RPMI 1640 (shown in the leftmost sketch). The cell culture medium was harvested 24 h later for cytokine measurement (*n* = 3 per group). *P < 0.05, **P < 0.01; statistical significance was analyzed using unpaired Student’s *t* test.

We hypothesized that high levels of bilirubin, in addition to directly inducing apoptosis in hepatocytes and neuronal cells, may act as a danger signal that triggers an inflammatory response in the liver. To test these hypotheses, we treated HepG2 and SynHeps-II cells with increasing concentrations of bilirubin and monitored cell death under a fluorescence microscope using a TUNEL (terminal deoxynucleotidyl transferase-mediated deoxyuridine triphosphate nick end labeling) staining kit (Fig. 7A). It was found that bilirubin at concentrations of 50 μM and above significantly induced apoptosis in HepG2 cells, but the effect was much weaker in SynHeps-II cells (Fig. 7A). Considering the scenario of hepatic encephalopathy, we also tested and found the toxic effects of high concentrations of bilirubin on brain neuronal cell HT22 and astrocyte U251 (Fig. 7B). To test the possibility of bilirubin-induced inflammation, PBMCs were incubated with increasing concentrations of bilirubin for 24 h, and the activation of NF-κB, one of the major proinflammatory signaling pathways, was analyzed (Fig. 7C). The results showed that NF-κB was apparently activated by bilirubin at concentrations of 50 μM and above (Fig. 7C), as indicated by the elevated phosphorylation of NF-κB p65 (Fig. S4A). Furthermore, PBMCs secreted more proinflammatory cytokines IL-1β, IL-6, TNF-α, and IL-12 after being coincubated with increasing concentrations of bilirubin (Fig. S4B). Supplementation of SynHeps-II cells in the PBMCs-bilirubin coincubation system significantly reduced the production of proinflammatory cytokines IL-1β, IL-6, TNF-α, and IL-12 by PBMCs (Fig. 7D). To sum up, high levels of bilirubin not only induced cellular apoptosis in hepatocytes and neuronal cells but also triggered an inflammatory response in immune cells.

### High concentrations of ammonia raised intracellular ROS level and activated neutrophils

**Fig. 8.**
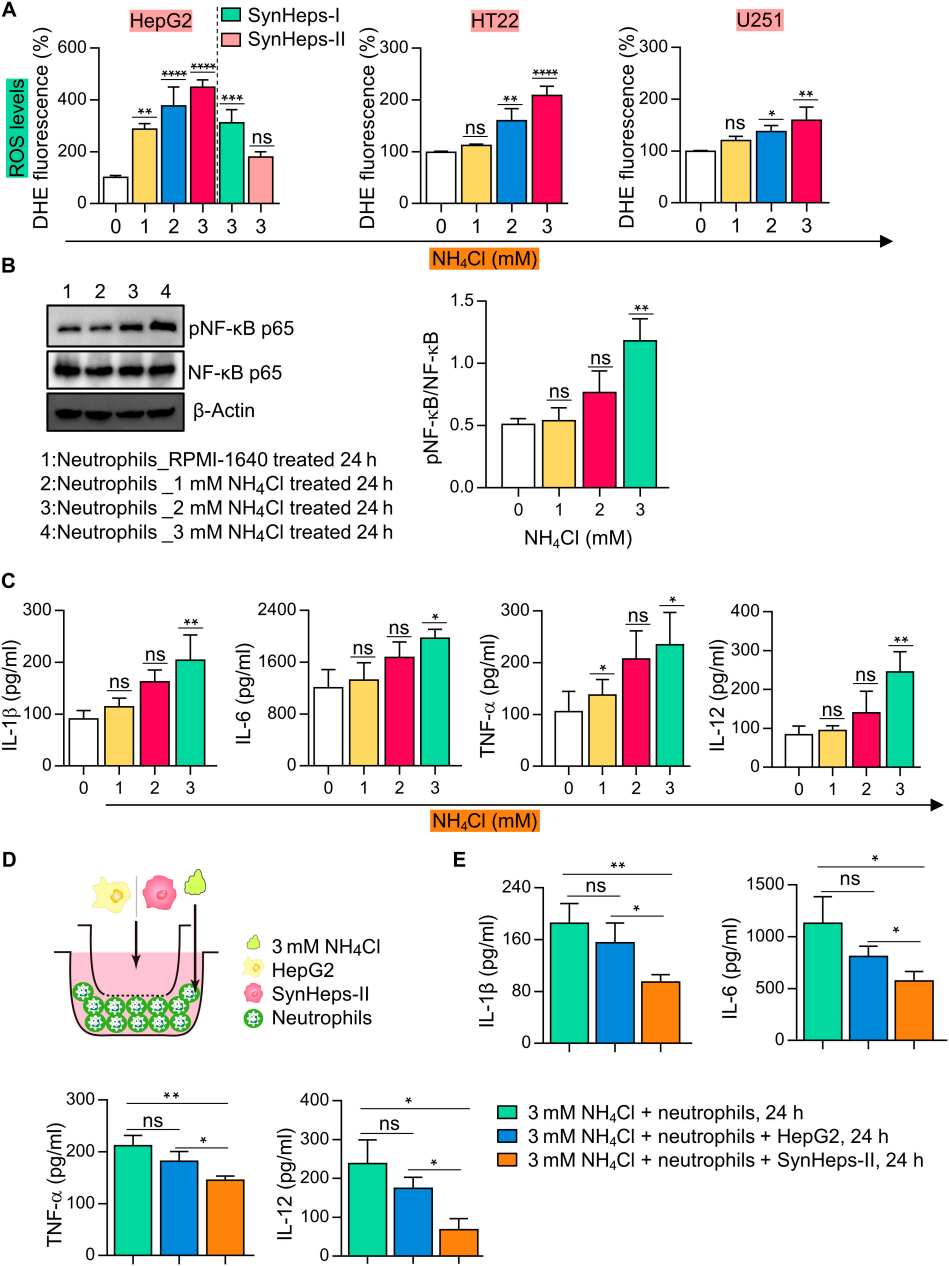
High concentration of ammonia raised intracellular ROS level and activated neutrophils. (A) HepG2, SynHeps-I, SynHeps-II, HT22, and U251 cells were inoculated into 24-well plates at 0.2 × 10^6^ cells per well, and after the cells were attached and treated with different concentrations of NH_4_Cl for 24 h, the ROS levels in the cell mixture were measured (*n* = 3 per group). DHE fluorescence intensity relative to the control group (0 mM NH_4_Cl treatment) was quantified (*n* = 3). *P < 0.05, **P < 0.01, ***P < 0.001, ****P < 0.0001; statistical significance was analyzed by one-way ANOVA. (B) Western blot analysis of NF-κB signaling molecules NF-κB p65 and phosphorylated NF-κB p65. Neutrophils were incubated with increasing concentrations of NH_4_Cl for 24 h. Cell lysate was prepared, separated on SDS-PAGE, and probed with corresponding antibodies. Band intensity in the Western blot was further quantified (right panel, *n* = 3 per group). Total protein was used for normalization. *P < 0.05, **P < 0.01; statistical significance was analyzed using unpaired Student’s *t* test. (C) Secretion of proinflammatory cytokines by neutrophils after NH_4_Cl stimulation. Neutrophils were inoculated with increasing concentrations of ammonia for 24 h. The cell culture medium was collected and analyzed for cytokine (IL-1β, IL-6, TNF-α, and IL-12) concentrations by ELISA (*n* = 3 per group). *P < 0.05, **P < 0.01; statistical significance was analyzed by one-way ANOVA. (D) Schematic diagram of neutrophils being cocultured with HepG2/SynHeps-II. Neutrophils were seeded in 24-well tissue culture plate at 1 × 10^6^ cells per well, and then HepG2/SynHeps-II cells were seeded at 1 × 10^6^ cells per well in the Transwell. Then, 3 mM NH_4_Cl was added to the 24-well plate and cells were cocultured in RPMI 1640 for 24 h. (E) Concentrations of IL-1β, IL-6, TNF-α, and IL-12 from the cocultured medium measured by ELISA (*n* = 3 per group). *P < 0.05, **P < 0.01; statistical significance was analyzed using unpaired Student’s *t* test.

As mentioned before, there was ammonia accumulation in the CSF (Fig. 5I) and high ROS levels in the brain of ALF mice (Fig. 5R and S). To test whether a high concentration of ammonia could cause the generation of excessive ROS, we incubated different cells (HepG2, SynHeps-I, SynHeps-II, HT22, and U251) with NH_4_Cl for 24 h and found that the intracellular ROS levels indeed increased with the existence of high ammonia concentrations (Fig. 8A). Both ammonia and ROS have been regarded as the key implicating factors in hepatic encephalopathy [12]. Here, we found that high concentrations of ammonia directly activated neutrophils through NF-κB signaling (Fig. 8B and C). When isolated neutrophils from donors were incubated with a high concentration of NH_4_Cl for 24 h, phosphorylation of NF-κB p65 (Fig. 8B) and secretion of proinflammatory cytokines IL-1β, IL-6, TNF-α, and IL-12 (Fig. 8C) was significantly increased with the increment of ammonia concentration. We then tested whether SynHeps-II cell could reduce the activation of neutrophils induced by a high concentration of ammonia. After supplementation of SynHeps-II cells to the mixed culture of neutrophils and NH_4_Cl that were separated from SynHeps-II cells by permeable membrane (Fig. 8D), the production of proinflammatory cytokines IL-1β, IL-6, TNF-α, and IL-12 was significantly reduced (Fig. 8E). Taken together, a high concentration of ammonia could raise the intracellular ROS level and directly activate neutrophils, which makes ammonia and neutrophils as potential therapeutic targets for ALF.

## Discussion

Bilirubin and ammonia are the major forms of serum toxic metabolite accumulated in liver failure disease. High levels of bilirubin and ammonia are toxic to brain, and the latter can cause hepatic encephalopathy. Both bilirubin and ammonia are strongly associated with mortality in patients with liver failure, and reduction of bilirubin and ammonia concentrations to normal levels tends to have a favorable prognosis [9,31]. Previous BAL (e.g., ELAD) used the HepG2-derived cell line C3A as working cells and had been tested in clinical trials [32,33]. However, ELAD failed to improve survival rate in severe alcoholic hepatitis patients [13]. Mechanistically, it was skeptical whether ELAD could at least partially undertake the metabolic functions of the injured liver since C3A cells lack the functions of ammonia metabolism and the parental HepG2 cells have very low expression of UGT1A1 (Fig. 2B and C), which was the only essential gene for bilirubin detoxification. In the present study, HepG2-derived SynHeps-II cells exhibited superior urea conversion, synthesizing twice as much urea as C3A cells and nearly half as much as normal human primary hepatocytes [19,21]. In contrast to the inability of C3A to metabolize ammonia [14,34] and bilirubin [13], SynHeps-II cells were efficient to detoxify ammonia, reducing ammonia in ALF patient plasma from approximately 1,000 μM to 450 μM in vitro (Fig. 2H), and reduced the plasma bilirubin from approximately 80 μM to 25 μM (Fig. 2G). Interestingly, SynHeps-I and SynHeps-II cells appeared to be more energetic compared to parental HepG2 cells, as evidenced by increased oxygen and sugar consumption (Fig. 2I). While SynHeps-I cells were effective in the treatment of ALF mice (Fig. 5B) and rabbits (Fig. 6B), the efficacy of SynHeps-II cells was more pronounced, with an increase in survival rate by approximately 30% in both cases, suggesting that elimination of ammonia in addition to bilirubin was necessary for maximal efficacy. Similarly, a previous study also incorporated bilirubin pre-adsorption before BAL treatment of ALF pigs using primary pig hepatocytes, and the result was satisfactory in improving the overall survival rate of the experimental animals [19].

We hypothesized that a high concentration of bilirubin may serve as a DAMPs molecule that triggered systemic inflammatory response. In support of this, it was found that bilirubin at 50 μM and above not only caused damage to hepatocytes and neuronal cells (Fig. 7A and B) but also activated PBMCs through the NF-κB pathway (Fig. 7C). This finding was consistent with previous findings that free bilirubin had neurotoxicity [35,36]. Engineering of metabolic pathways to detoxify bilirubin has received little attentions in previous BAL studies. Only a few bilirubin adsorption resins have been used for MAL devices, and the efficiency was limited due to the absorbing capacity and specificity of the materials, typically resulting in a reduction rate of bilirubin by about 35% or less [37]. Our current PCC-BAL could reduce the serum bilirubin by approximately 70% in vitro (Figs. 2G and 4H) and reduce the serum bilirubin to normal physiological levels in the ALF rabbit models (Fig. 6E), which represented a significant advancement.

In addition to causing hyperbilirubinemia, ALF also leads to hyperammonemia, inflammatory responses, and oxidative stress, which together contribute to the development of hepatic encephalopathy [38]. ALF-induced hepatic encephalopathy is mainly due to the impaired functioning of hepatocytes in the urea cycle, resulting in the excess ammonia reflux into the brain. This causes astrocytes to metabolize excessive amounts of ammonia, which leads to cellular edema and even necrosis, resulting in neuronal cells being unprotected as well, which ultimately lead to excessive cellular production of ROS, and triggers an inflammatory response in the brain, a process that may be accompanied by neutrophil activation (Fig. 8B). Moreover, unconjugated bilirubin can also cross the blood–brain barrier, causing damage to astrocytes and neuronal cells, and synergizes with cerebral ammonia to cause SIRS, increasing the risk of MOF.

Macrophages play a key role in ALF-triggered SIRS. Liver-resident macrophages (known as Kupffer cells) account for up to 80% of human macrophages [5]. Following liver injury, stimuli [e.g., pathogen-associated molecular patterns (PAMPs) and DAMPs] released by damaged cells act on Kupffer cells, which subsequently release chemokines to recruit monocytes that will differentiate into monocyte-derived macrophages (MoMϕs) in the liver [39,40]. Both Kupffer cells and MoMϕs contribute to the progression of inflammation and injury in liver diseases [39,40]. Besides, we found that a high concentration of ammonia also induced neutrophil activation, leading to the release of a range of proinflammatory factors that exhibited brain edema and inflammation (Figs. 5O and P and 8B to D), while brain inflammation and ammonia also affect brain vascular dysfunction and leakage (Fig. 5T and U). Therefore, elimination of proinflammatory mediators contributes to liver regeneration, as well as to the mitigation of the development of hepatic encephalopathy.

The process of liver recovery is accompanied by liver regeneration and elimination of the inflammatory status, which are regulated by STAT3/STAT1 [41,42] and inflammatory NF-κB signaling pathways [43], respectively. By studying histology and immunohistochemistry (Figs. 5K and 6G), and measuring blood proinflammatory cytokines (Fig. 6F and Fig. S3D) and liver HGF and Ki67 expression levels (Figs. 5K and 6G), it was demonstrated that treatment with SynHeps-I and SynHeps-II cells significantly ameliorated the proinflammatory state of the liver in both ALF mouse and rabbit models by inhibiting NF-κB signaling (Fig. 5J), suppressing CD86 expression in the liver, suppressing CD11b expression in the brain (Fig. 5M and P), and reducing the levels of proinflammatory cytokines in the blood (Fig. 6F and Fig. S3D). Immunofluorescence staining analysis of liver tissues showed that liver recovery after ALF was a dynamic process, influenced by liver regeneration (represented by Cy3-labeled HGF staining) and liver injury (represented by Alexa Fluor 488-labeled P21 staining). In Cytopore_SynHeps-II-treated mice, HGF expression was high on day 3, and the expression of P21 was also elevated (but lower than that in the Cytopore_HepG2 group). After treatment on day 7, there was still a small amount of HGF expression, but the expression of P21 had basically disappeared, which indicated that the liver injury had been reversed and the liver had returned to the normal state (Fig. 5K). A similar dynamic trend was observed in ALF rabbit liver tissues, with the slight difference in that HGF continued to be expressed at high levels in rabbit liver tissues (Fig. 6G).

Despite the impressive efficacy of the SynHeps-II cell-based BAL in treating ALF animals, a few limitations of the current study should be acknowledged. Due to the limited space of the article, we have only systematically investigated the therapeutic effect of Cytopore_SynHeps-II cells on d-gal-induced ALF in mice and rabbits. Indeed, this study could be further investigated on other ALF models such as acetaminophen (APAP) and other drug-induced ALF, or ALF induced by surgical resection. Future directions of research for clinical applications should explore the benefit of SynHeps-II-based BAL monotherapy and in combination with currently available adjuvant treatments such as early renal replacement therapy and possibly therapeutic plasma exchange.

### Conclusion

Current study developed an innovated PCC-BAL system by targeting the bilirubin and ammonia metabolisms. The genetically engineered SynHeps-II cells were very effective in detoxifying bilirubin and ammonia in the blood and CSF. PCC-BAL-embedded SynHeps-II cell therapy could alleviate liver and brain inflammation, attenuate the damage of these organs, and improve liver microenvironment that benefits liver regeneration. Treatment of ALF rabbits with PCC-BAL saved 80% of animals from death. Taken together, PCC-BAL has clinical potentials for the treatment of ALF patients.

## Data Availability

Materials from the present study are available from the corresponding author on reasonable request.
