# Supplementary material for: Genetically Modified Hepatocytes Targeting Bilirubin and Ammonia Metabolism for the Construction of Bioartificial Liver System
Source: Biomater Res. 2024 Jul 15;28:0043. doi: 10.34133/bmr.0043 (PMC11246981; doi:10.34133/bmr.0043)
Supplement: Supplementary 1 — Figs. S1 to S4 Table S1 Movies S1 to S4 [file bmr.0043.f1.zip › Supplemental Material.docx]

**Supplementary materials**


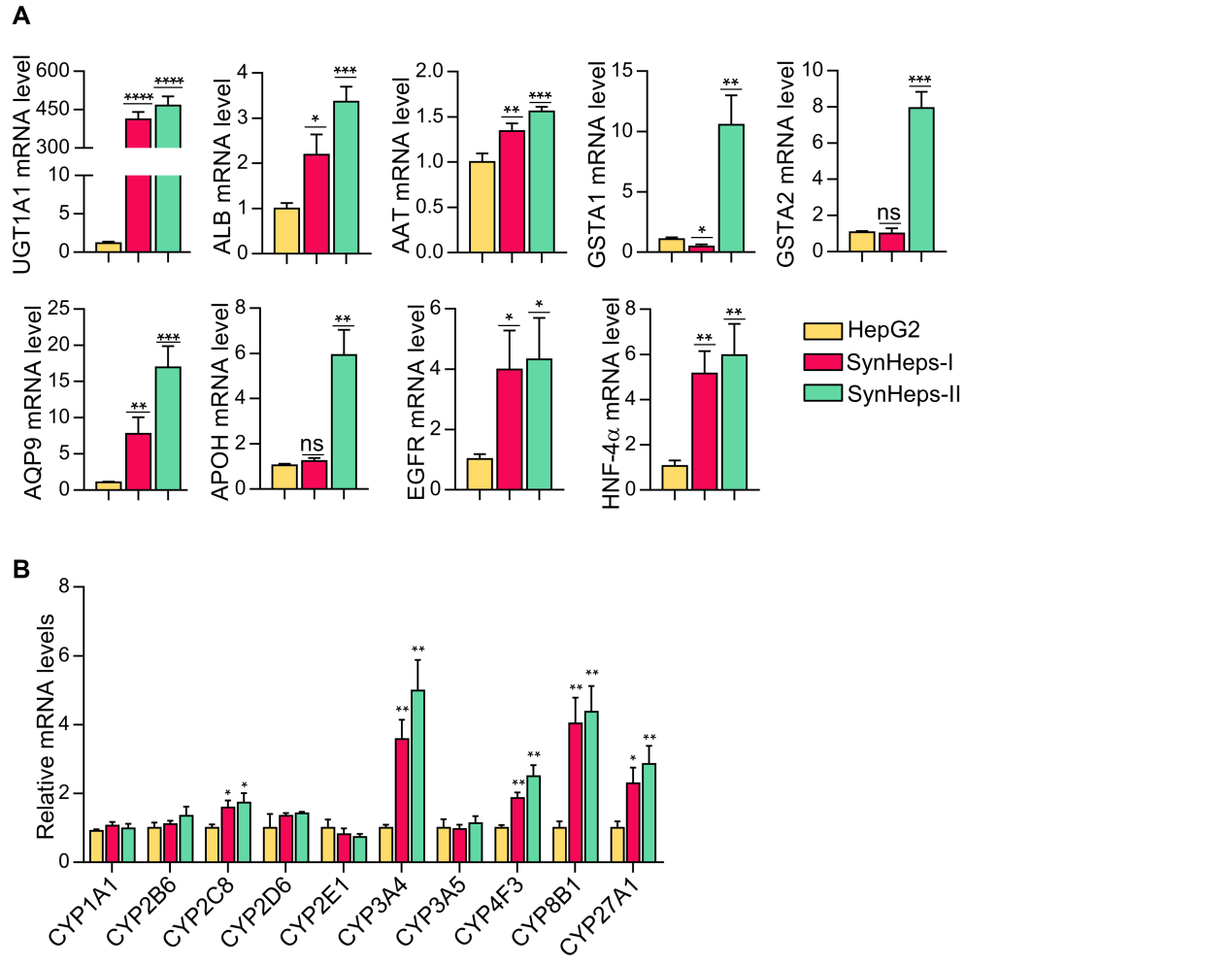


**Figure S1** Quantification of gene expression in HepG2, SynHeps-I and SynHeps-II cells by qPCR.

(**A**) Expression levels of UGT1A1, ALB, AAT, GSTA1/2, AQP9, APOH, EGFR and HNF-4α genes in HepG2, SynHeps-I and SynHeps-II cells. N=3, *p < 0.05, **p < 0.01, ***p < 0.001, ****p < 0.0001, ns, not significant, statistical significance was analyzed by one-way ANOVA. (**B**) Expression level of representative genes of the CYP450 gene family in HepG2, SynHeps-I and SynHeps-II cells. Quantification was performed by qPCR and HepG2 was used for normalization. N=3, *p < 0.05, **p < 0.01, ns, not significant, statistical significance was analyzed by one-way ANOVA.


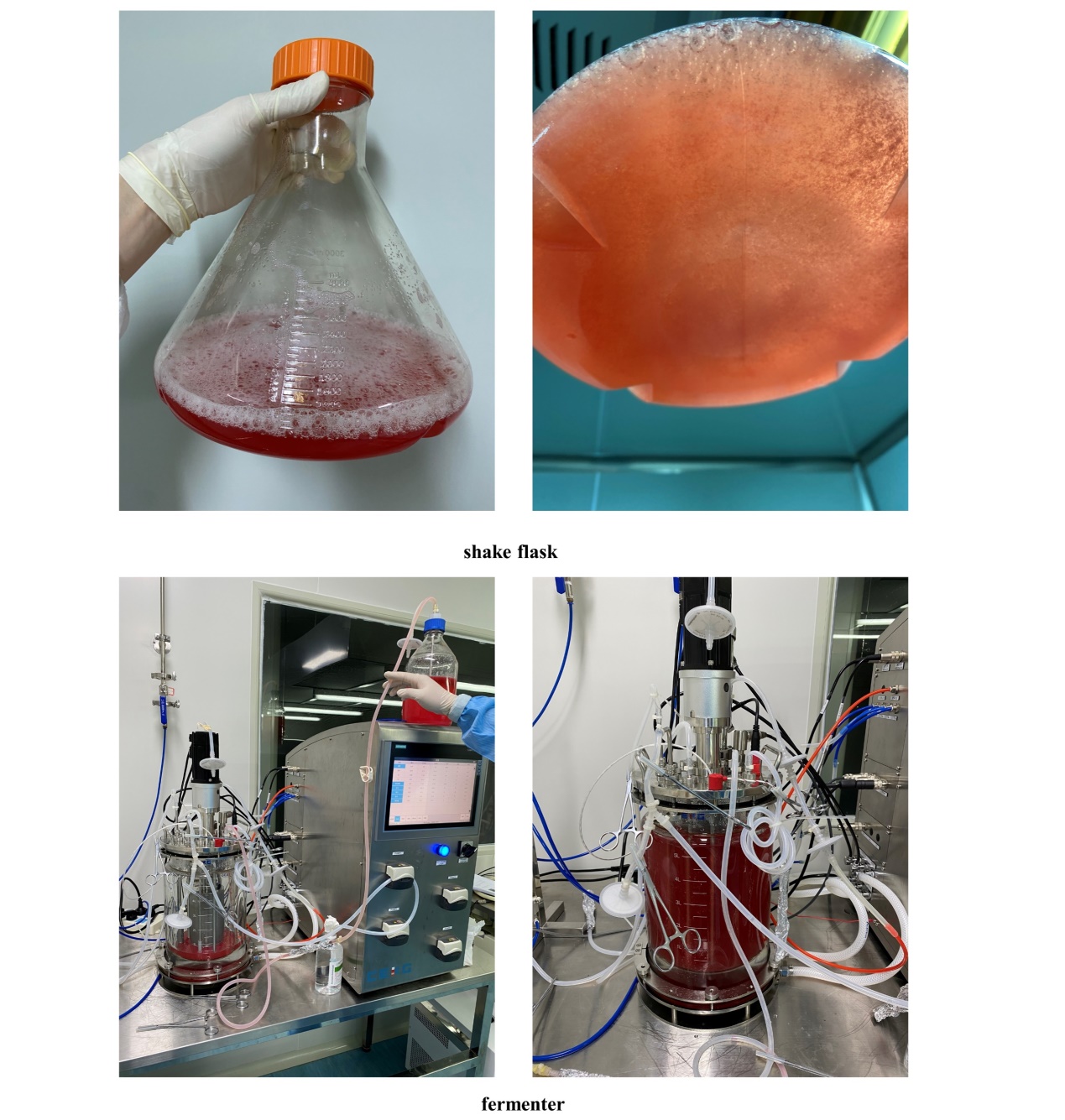


**Figure S2** Three-dimensional expansion of SynHeps cells in shake flask and fermenter.


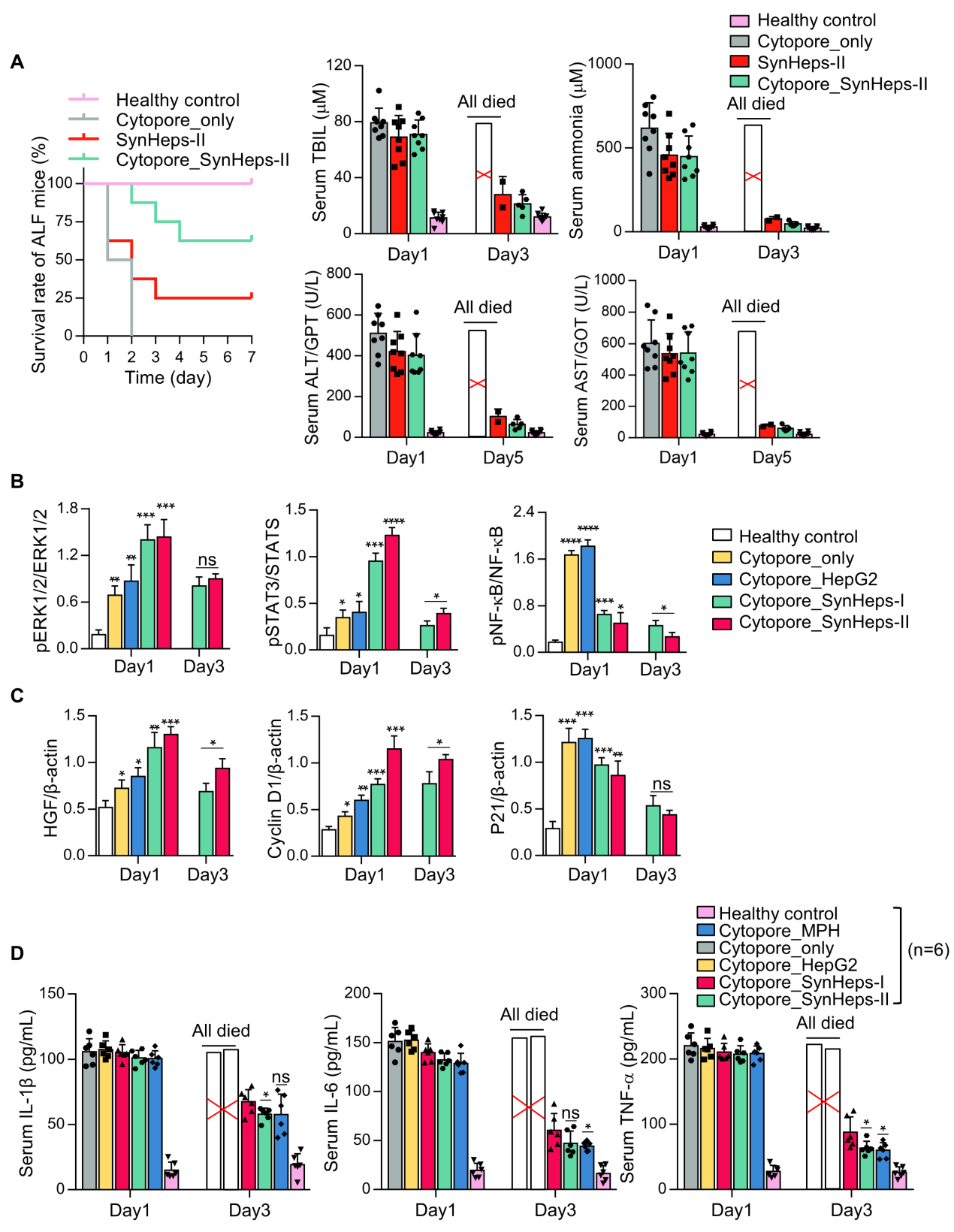


**Figure S3** Treatment of ALF mice with Cytopore-SynHeps-II cells.

**(A)** Survival rate of ALF mice in different treatment groups. Serum concentrations of TBIL and ammonia on day 1 and day3, and serum AST and ALT levels on day1 and day 5 after treatment were also measured. (**B**) Quantification of band intensity in pERK1/2, pSTAT3 and pNF-κB p65 from Fig. 5J. Total protein was used for normalization. N=3, *P< 0.05, **P< 0.01, ***p < 0.001, ****p < 0.0001, ns, not significant, statistical significance was analyzed using unpaired student t-test. (**C**) Quantification of band intensity in HGF, Cyclin D1 and P21 from Fig. 5J. β-actin was used for normalization. N=3, *P< 0.05, **P< 0.01, ***p < 0.001, ns, not significant, statistical significance was analyzed using unpaired student t-test. (**D**) Serum levels of pro-inflammatory cytokines IL-1β, IL-6, and TNF-α in healthy control and ALF mice on day 1 and day 3 after treatment with Cytopore_only, Cytopore_cells, and Cytopore_MPH. On day 3, the Cytopore_SynHeps-II and Cytopore_MPH groups were statistically analyzed against the Cytopore_SynHeps-I group. *p < 0.05, ns, not significant, statistical significance was analyzed using unpaired student t-test.


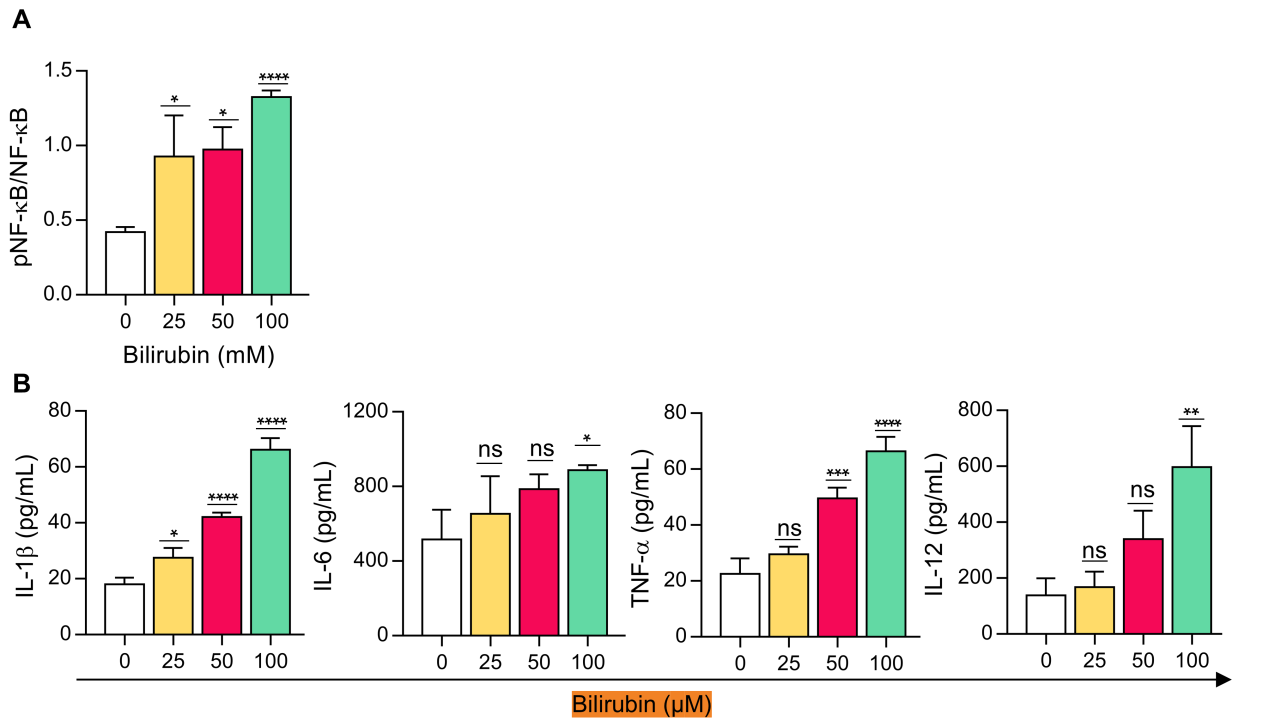


**Figure S4** Activation of PBMC by high concentrations of bilirubin.

(**A**) Quantitative analysis of pNF-κB from Fig. 7C. Total protein was used for normalization. N=3, *P< 0.05, ****P< 0.0001, ns, not significant, statistical significance was analyzed using unpaired student t-test. (**B**) Secretion of pro-inflammatory cytokines by PBMC after bilirubin stimulation. PBMC cells were incubated with increasing concentrations of bilirubin for 24 hours. Cell culture media was collected and concentrations of IL-1β, IL-6, and TNF-α were measured by ELISA (n=3 per group). *p< 0.05, **p< 0.01, ***p< 0.001, ****P< 0.0001, ns, not significant, statistical significance was analyzed by one-way ANOVA.

**Movie S1** ALF mice with concomitant signs of hepatic encephalopathy.

D-gal induced acute liver failure in mice was accompanied by hepatic encephalopathy. The mice showed anorexia, unsteady movement, inactivity, and delayed response to external stimuli, etc.

**Movie S2** Behavior of rabbits with D-gal-induced ALF.

Twenty hours later after intraperitoneal injection of D-gal, rabbits appeared anorexic, comatose, and insensitive to pain and external stimuli.

**Movie S3** Treatment of ALF rabbits with the PCC-BAL system.

The rabbit's life signs remained stable during the treatment.

**Movie S4** Recovery of ALF rabbits after PCC-BAL treatment.

After treatment with the PCC-BAL system, the ALF rabbits recovered well with stable life signs, regained mobility, ate normally, and responded normally to external stimuli.

**Table S1** Real-time PCR primers used in the current study.

| Gene | Forward: 5’-3’ | Reverse: 5’-3’ |
| --- | --- | --- |
| GAPDH | **F:** GCACCGTCAAGGCTGAGAAC | **R:** TGGTGAAGACGCCAGTGGA |
| ALB | **F:** GCCTTTGCTCAGTATCTT | **R:** AGGTTTGGGTTGTCATCT |
| AAT | **F:** TATGATGAAGCGTTTAGGC | **R:** CAGTAATGGACAGTTTGGGT |
| UGT1A1 | **F:** CAGAACTTTCTGTGCGACGTG | **R:** GGGTAATCCTTCACAAAGTC |
| CYP1A1 | **F:** ACCAGGACCCTGTCCAATCT | **R:** GAAGGCAGCCCTGTTTGTTC |
| CYP2B6 | **F:** TCTGGCCGGGGAAAAATCG | **R:** GGTCACAGAGAATCGCCGAAG |
| CYP2C8 | **F:** GGAAAACGAATTTGTGCAGGAG | **R:** GTGGCAGAGAAACAATCCCTT |
| CYP2D6 | **F:** CCAACGGTCTCTTGGACAAAG | **R:** GGGTCGTCGTACTCGAAGC |
| CYP2E1 | **F:** GATGCCCTACATGGATGCTG | **R:** AAATGGTGTCTCGGGTTGCT |
| CYP3A4 | **F:** GTGGGGCTTTTATGATGGTC | **R:** CATCTCCATACTGGGCAATGA |
| CYP3A5 | **F:** GCAAACAGCCCAGCAAACA | **R:** GTCCATCGCCACTTTCCTTC |
| CYP4F3 | **F:** AAGATGGGAAGAAGTTGTC | **R:** CCTCTCCTTGATGTTCTTTG |
| CYP8B1 | **F:** TCATTGCTGGATACCTGTGC | **R:** GTCCATGACGAAGGTGAAG |
| CYP27A1 | **F:** AAGCGATACCTGGATGGTTG | **R:** TGTTGGATGTCGTGTCCACT |
| HNF-4α | **F:** ACAGATGTCCACCCCTGAGA | **R:** AGAGGGGCTTGACGATTGTG |
| AQP9 | **F:** CTCCTGATTATTGTCATTG | **R:** ATCCACCAGAAGTTGTTT |
| APOH | **F:** TGCTATTGCAGGACGGACCT  TGCTATTGCAGGACGGACCT  CTTCCAGTTCCCGCTATGCTA  CTTCCAGTTCCCGCTATGCTA  CTTCCAGTTCCCGCTATGCTA  CTTCCAGTTCCCGCTATGCTA  CTTCCAGTTCCCGCTATGCTA | **R:** GCTCATAGAATGTTTTTAACG |
| GSTA1 | **F:** CTGCCCGTATGTCCACCTG | **R:** TCAAAGGCAGGGAAGTAGC |
| GSTA2 | **F:** CAGTAACCTGCCCACAGTGAAG | **R:** CATGTTCTTGACCTCTATGGCTG |
| EGFR | **F:** TAACAAGCTCACGCAGTTGG | **R:** GTTGAGGGCAATGAGGACAT |
| Bax | **F:** AGAGGATGATTGCCGCCGT | **R:** CAACCACCCTGGTCTTGGAT |
| Bcl-2 | **F:** CCTGTGGATGACTGAGTACC | **R:** GAGACAGCCAGGAGAAATCA |
