# Supplementary material for: Genetically Modified Hepatocytes Targeting Bilirubin and Ammonia Metabolism for the Construction of Bioartificial Liver System
Source: Biomater Res. 2024 Jul 15;28:0043. doi: 10.34133/bmr.0043 (PMC11246981; doi:10.34133/bmr.0043)
Supplement: Supplementary 1 — Figs. S1 to S4 Table S1 Movies S1 to S4 [file bmr.0043.f1.zip › Table S1.docx]

**Table S1**

| Gene | Forward: 5’-3’ | Reverse: 5’-3’ |
| --- | --- | --- |
| GAPDH | **F:** GCACCGTCAAGGCTGAGAAC | **R:** TGGTGAAGACGCCAGTGGA |
| ALB | **F:** GCCTTTGCTCAGTATCTT | **R:** AGGTTTGGGTTGTCATCT |
| AAT | **F:** TATGATGAAGCGTTTAGGC | **R:** CAGTAATGGACAGTTTGGGT |
| UGT1A1 | **F:** CAGAACTTTCTGTGCGACGTG | **R:** GGGTAATCCTTCACAAAGTC |
| CYP1A1 | **F:** ACCAGGACCCTGTCCAATCT | **R:** GAAGGCAGCCCTGTTTGTTC |
| CYP2B6 | **F:** TCTGGCCGGGGAAAAATCG | **R:** GGTCACAGAGAATCGCCGAAG |
| CYP2C8 | **F:** GGAAAACGAATTTGTGCAGGAG | **R:** GTGGCAGAGAAACAATCCCTT |
| CYP2D6 | **F:** CCAACGGTCTCTTGGACAAAG | **R:** GGGTCGTCGTACTCGAAGC |
| CYP2E1 | **F:** GATGCCCTACATGGATGCTG | **R:** AAATGGTGTCTCGGGTTGCT |
| CYP3A4 | **F:** GTGGGGCTTTTATGATGGTC | **R:** CATCTCCATACTGGGCAATGA |
| CYP3A5 | **F:** GCAAACAGCCCAGCAAACA | **R:** GTCCATCGCCACTTTCCTTC |
| CYP4F3 | **F:** AAGATGGGAAGAAGTTGTC | **R:** CCTCTCCTTGATGTTCTTTG |
| CYP8B1 | **F:** TCATTGCTGGATACCTGTGC | **R:** GTCCATGACGAAGGTGAAG |
| CYP27A1 | **F:** AAGCGATACCTGGATGGTTG | **R:** TGTTGGATGTCGTGTCCACT |
| HNF-4α | **F:** ACAGATGTCCACCCCTGAGA | **R:** AGAGGGGCTTGACGATTGTG |
| AQP9 | **F:** CTCCTGATTATTGTCATTG | **R:** ATCCACCAGAAGTTGTTT |
| APOH | **F:** TGCTATTGCAGGACGGACCT  TGCTATTGCAGGACGGACCT  CTTCCAGTTCCCGCTATGCTA  CTTCCAGTTCCCGCTATGCTA  CTTCCAGTTCCCGCTATGCTA  CTTCCAGTTCCCGCTATGCTA  CTTCCAGTTCCCGCTATGCTA | **R:** GCTCATAGAATGTTTTTAACG |
| GSTA1 | **F:** CTGCCCGTATGTCCACCTG | **R:** TCAAAGGCAGGGAAGTAGC |
| GSTA2 | **F:** CAGTAACCTGCCCACAGTGAAG | **R:** CATGTTCTTGACCTCTATGGCTG |
| EGFR | **F:** TAACAAGCTCACGCAGTTGG | **R:** GTTGAGGGCAATGAGGACAT |
| Bax | **F:** AGAGGATGATTGCCGCCGT | **R:** CAACCACCCTGGTCTTGGAT |
| Bcl-2 | **F:** CCTGTGGATGACTGAGTACC | **R:** GAGACAGCCAGGAGAAATCA |
